# Assessment of Hypertension Complications and Health Service Use 5 Years After Implementation of a Multicomponent Intervention

**DOI:** 10.1001/jamanetworkopen.2023.15064

**Published:** 2023-05-24

**Authors:** Esther Y. T. Yu, Eric Y. F. Wan, Ivy L. Mak, David V. K. Chao, Welchie W. K. Ko, Maria Leung, Yim Chu Li, Jun Liang, Wan Luk, Michelle M. Y. Wong, Tony K. H. Ha, Anca K. C. Chan, Daniel Y. T. Fong, Cindy L. K. Lam

**Affiliations:** 1 Department of Family Medicine and Primary Care, School of Clinical Medicine, Li Ka Shing Faculty of Medicine, The University of Hong Kong, Hong Kong SAR, China; 2 Department of Family Medicine and Primary Care, The University of Hong Kong–ShenZhen Hospital, Shen Zen, China; 3 Centre for Safe Medication Practice and Research, Department of Pharmacology and Pharmacy, Li Ka Shing Faculty of Medicine, The University of Hong Kong, Hong Kong SAR, China; 4 Laboratory of Data Discovery for Health (D24H), Hong Kong Science and Technology Park, Sha Tin, Hong Kong SAR, China; 5 Department of Family Medicine, Kowloon East Cluster, Hong Kong Hospital Authority, Hong Kong SAR, China; 6 Department of Family Medicine, Hong Kong West Cluster, Hong Kong Hospital Authority, Hong Kong SAR, China; 7 Department of Family Medicine, New Territories East Cluster, Hong Kong Hospital Authority, Hong Kong SAR, China; 8 Department of Family Medicine, Kowloon Central Cluster, Hong Kong Hospital Authority, Hong Kong, China; 9 Department of Family Medicine, New Territories West Cluster, Hong Kong Hospital Authority, Hong Kong, China; 10 Department of Family Medicine, Kowloon West Cluster, Hong Kong Hospital Authority, Hong Kong SAR, China; 11 Department of Family Medicine, Hong Kong East Cluster, Hong Kong Hospital Authority, Hong Kong, China; 12 Hospital Authority Head Office, Hong Kong Hospital Authority, Hong Kong SAR, China; 13 New Territories East Cluster Statistics Office, Prince of Wales Hospital, Hong Kong Hospital Authority, Hong Kong SAR, China; 14 School of Nursing, Li Ka Shing Faculty of Medicine, The University of Hong Kong SAR, China

## Abstract

**Importance:**

There is a lack of information regarding the impact of implementing a protocol-driven, team-based, multicomponent intervention in public primary care settings on hypertension-related complications and health care burden over the long term.

**Objective:**

To compare hypertension-related complications and health service use at 5 years among patients managed with Risk Assessment and Management Program for Hypertension (RAMP-HT) vs usual care.

**Design, Setting, and Participants:**

In this population-based prospective matched cohort study, patients were followed up until the date of all-cause mortality, an outcome event, or last follow-up appointment before October 2017, whichever occurred first. Participants included 212 707 adults with uncomplicated hypertension managed at 73 public general outpatient clinics in Hong Kong between 2011 and 2013. RAMP-HT participants were matched to patients receiving usual care using propensity score fine stratification weightings. Statistical analysis was conducted from January 2019 to March 2023.

**Interventions:**

Nurse-led risk assessment linked to electronic action reminder system, nurse intervention, and specialist consultation (as necessary), in addition to usual care.

**Main Outcomes and Measures:**

Hypertension-related complications (cardiovascular diseases, end-stage kidney disease), all-cause mortality, public health service use (overnight hospitalization, attendances at accident and emergency department, specialist outpatient clinic, and general outpatient clinic).

**Results:**

A total of 108 045 RAMP-HT participants (mean [SD] age: 66.3 [12.3] years; 62 277 [57.6%] female) and 104 662 patients receiving usual care (mean [SD] age 66.3 [13.5] years; 60 497 [57.8%] female) were included. After a median (IQR) follow-up of 5.4 (4.5-5.8) years, RAMP-HT participants had 8.0% absolute risk reduction in cardiovascular diseases, 1.6% absolute risk reduction in end-stage kidney disease, and 10.0% absolute risk reduction in all-cause mortality. After adjusting for baseline covariates, the RAMP-HT group was associated with lower risk of cardiovascular diseases (hazard ratio [HR], 0.62; 95% CI, 0.61-0.64), end-stage kidney disease (HR, 0.54; 95% CI, 0.50-0.59), and all-cause mortality (HR, 0.52; 95% CI, 0.50-0.54) compared with the usual care group. The number needed to treat to prevent 1 cardiovascular disease event, end-stage kidney disease, and all-cause mortality was 16, 106, and 17, respectively. RAMP-HT participants had lower hospital-based health service use (incidence rate ratios ranging from 0.60 to 0.87) but more general outpatient clinic attendances (IRR, 1.06; 95% CI, 1.06-1.06) compared with usual care patients.

**Conclusions and Relevance:**

In this prospective matched cohort study involving 212 707 primary care patients with hypertension, participation in RAMP-HT was associated with statistically significant reductions in all-cause mortality, hypertension-related complications, and hospital-based health service use after 5 years.

## Key Points

**Question:**

Is a protocol-driven, team-based, multicomponent hypertension management program implemented in the public primary care setting associated with fewer complications and mortality in patients with hypertension?

**Findings:**

In this cohort study that included 212 707 adults with uncomplicated hypertension, participation in the Risk Assessment and Management Program for Hypertension, offered in addition to usual primary care, was associated with clinically significant reductions in incidences of hypertension-related complications, all-cause mortality, and hospital-based health service use after 5 years.

**Meaning:**

This study’s results suggest that a protocol-driven, team-based hypertension management program incorporated into the public primary care setting is a viable strategy to reduce disease burden on health care systems due to hypertension.

## Teaser

This cohort study investigates hypertension-related complications and health service use at 5 years among patients who participated in a multicomponent intervention designed to improve the quality of hypertension management in the primary care setting in Hong Kong.

## Introduction

Hypertension is the leading global risk factor for mortality and morbidity resulting from coronary heart disease (CHD), stroke, and end-stage kidney disease (ESKD).^1^ One study^2^ found that only 1 in 3 patients receiving treatment for hypertension worldwide achieved blood pressure (BP) control. Team-based care, standardized management protocols, clinician training, and patient empowerment have shown effectiveness for BP reduction,^3,4,5^ whereas multilevel, multicomponent interventions have been most effective for lowering systolic BP (SBP).^6^ However, the effect of implementing these strategies on the population level is unclear, and evidence for longer-term effects (>24 months) on cardiovascular outcomes, mortality, or health service use remains sparse.

Hong Kong has a dual health care system, with a universal-type public system providing more than 90% of hospital-based services, 70% of chronic disease care, and approximately 30% of acute episodic care.^7^ The public system is heavily strained due to an aging population, increasing prevalence of chronic diseases, and limited manpower.^8^ Recent studies have found that, on average, each public outpatient clinic consultation lasted for 3 to 5 minutes,^9^ while each patient presented with 1.3 health problems including both new complaints and chronic diseases.^10^ Consequently, limited time can be spent on chronic disease care, contributing to therapeutic inertia among clinicians, poor patient-clinician communication, and patients’ nonadherence to treatment due to paucity of education and structured support.

According to an internal report, more than 45% of patients with hypertension receiving public primary care in Hong Kong had uncontrolled BP. To improve the quality of hypertension care, the territory-wide Risk Assessment and Management Program for Hypertension (RAMP-HT) was launched in 2011 by the Hospital Authority, the organization that manages public health services in Hong Kong.^11^ The RAMP-HT is a multilevel, multicomponent, team-based intervention, targeting total cardiovascular disease (CVD) risk control of primary care patients with uncomplicated hypertension, offered in addition to usual physician-led care. Three specific services were offered: (1) protocol-driven, nurse-led risk assessment and risk-stratified management linked to electronic action reminder system; (2) nurse intervention for patient empowerment; and (3) specialist consultations to manage resistant hypertension.

Preliminary results found that RAMP-HT participants with suboptimal BP at baseline were more likely to achieve target BP (odds ratio [OR], 1.18) and low-density lipoprotein cholesterol (LDL-C) levels (OR, 1.13), along with modest decreases in SBP, LDL-C, and predicted 10-year CVD risk compared with usual care patients after 12 months.^12^ It remains uncertain whether short-term reduction of these surrogate CVD markers translates to morbidity and mortality reduction over time. This study aimed to compare hypertension-related complications and health service use at 5 years among patients with hypertension managed with RAMP-HT vs usual care.

## Methods

### Study Design

This territory-wide prospective matched cohort study compared the risks of CVD, ESKD, all-cause mortality, and frequencies of public health service use after 5 years between RAMP-HT participants and patients with hypertension receiving usual public primary care in Hong Kong. This study followed the Strengthening the Reporting of Observational Studies in Epidemiology (STROBE) reporting guideline.

### Ethics

The research protocol was approved by the Clinical and Research Ethics Committees of all 7 Hospital Authority geographical clusters in Hong Kong. Anonymous data were extracted through the Hospital Authority–Clinical Management System (HA-CMS); thus, informed consent from participants was not required in accordance with 45 CFR §46.

### Settings

#### RAMP-HT

The setting and workflow of RAMP-HT has been described in detail previously^11,12^ and in eMethods 1 in Supplement 1. In brief, RAMP-HT introduced 3 specific services to augment usual care: nurse-led risk assessment, nurse intervention, and specialist consultation. The risk assessment was repeated every 12 to 30 months, whereas nurse intervention and specialist consultation were offered when necessary.

All participants underwent a standardized assessment on CVD risk, hypertensive complications, and self-care and were stratified into having low, medium, or high CVD risk according to the Joint British Societies’ (JBS2) risk calculator.^13^ The care-manager nurse then prepared a care plan and coordinated follow-up interventions for participants according to protocol (eFigure 1 in Supplement 1). The care plan was recorded on an electronic record platform with an action reminder system accessible at any public primary care clinic (ie, general outpatient clinic [GOPC]) to support team members’ clinical decision-making, including GOPC physicians providing usual care. Participants with adherence issues or specific risk factors were referred for nurse interventions, while patients with resistant hypertension were referred for additional specialist consultations. All participants continued to receive usual care every 8 to 16 weeks at GOPCs.

The RAMP-HT was intended for all adult patients with uncomplicated hypertension managed at GOPCs. However, due to the large number of patients, limited resources, and limited manpower, recruitment could only be performed in stages. Consequently, there was an opportunity window to compare the outcomes of RAMP-HT and usual care.

#### Usual Care

Patients who had not been enrolled in RAMP-HT continued to receive physician-led usual care at a GOPC every 8 to 16 weeks. During a typical consultation, the physician could review a patient’s BP and control of other risk factors, titrate medication, advise on lifestyle, arrange assessment, or refer to allied health professionals as appropriate, according to the Hong Kong reference framework for hypertension in primary care.^14^ Accesses to medications, laboratory tests, and allied health services were identical for both RAMP-HT and usual care patients.

### Study Participants

Patient inclusion criteria were (1) aged 18 years or older; (2) diagnosed with hypertension, defined by the International Classification of Primary Care, 2nd Edition (ICPC-2) code of K86; (3) not diagnosed with diabetes, CVD, or ESKD (defined in next section) on or before baseline; and (4) receiving hypertension care from any GOPCs. RAMP-HT participants were patients who enrolled in RAMP-HT between October 1, 2011, and September 30, 2013; usual care patients were those who had visited any GOPC at least once for hypertension care within the same period, but had not been enrolled in the RAMP-HT by September 30, 2017. Baseline dates for RAMP-HT and usual care groups were defined as the first date of attending a RAMP-HT risk assessment session and GOPC consultation during the aforementioned period, respectively. Each patient was observed from their baseline date to the date of all-cause mortality, incidence of an outcome event (defined in next section), or last follow-up censored until September 30, 2017, whichever occurred first.

### Outcome Measures

The primary composite outcome was any CVD events, including CHD, heart failure or stroke; ESKD; or all-cause mortality. Secondary outcomes included (1) CVD, (2) CHD, (3) stroke, (4) heart failure, (5) ESKD, (6) diabetes, (7) all-cause mortality, and (8) public health service use, including overnight hospitalization, and attendances at accident and emergency department, specialist outpatient clinic and GOPC. Events were defined based on ICPC-2 or *International Classification of Diseases, Ninth Revision, Clinical Modification* (eTable 1 in Supplement 1).

### Baseline Covariates

Baseline covariates included sociodemographics, medical history, anthropometric measurements, and laboratory results that were routinely collected at GOPC follow-ups; and these were listed in detail in eMethods 2 in Supplement 1. Sociodemographics included gender, age, and smoking status; medical history included the Charlson Comorbidity Index and medication prescriptions; anthropometric measurements included body weight, height, and BP; laboratory results included lipid profile, fasting glucose levels, and estimated glomerular filtration rate (eGFR) calculated with the abbreviated Modification of Diet in Renal Disease Study formula recalibrated for Chinese people.^15^

### Data Extraction

Outcome events, health service use, and characteristics of eligible patients from baseline up to September 30, 2017, were extracted from electronic health records from the Hospital Authority–Clinical Management System (HA-CMS) in September 2018. Mortality data were extracted from the Hong Kong Death Registry concurrently.

### Statistical Analysis

Missing baseline covariates were handled by multiple imputation. Missing values were imputed 5 times by chained equation method using all known baseline covariates and event outcomes.^16^ The same analysis was performed for each data set and the 5 sets of results were combined using Rubin rules.^17^

To reduce potential selection bias, all RAMP-HT participants and usual care patients were matched by propensity score–fine stratification weighting, which is an extension of propensity score matching that combines propensity score stratification with weighting technique.^18^ The propensity score for each patient was generated by fitting a logistic regression with the patient’s corresponding RAMP-HT or usual care group as a dependent variable and all baseline covariates as independent variables. The overall analytic sample was then stratified into 1000 quintiles of the propensity score, and a weight for each individual was generated based on their corresponding stratum and treatment assignment based on inverse probability of treatment weights.^19^ To apply this matching approach, the MMWS package in Stata was used.^20^

After weighting, summary statistics were described as mean (SD) or frequency (proportion). Balance of baseline covariates between the 2 groups was further assessed using standardized mean difference (SMD) to show the magnitude of difference in characteristics between groups. SMD of less than 0.2 implies sufficient balance between groups.^21^ To evaluate the changes in clinical parameters between baseline and 5 years, values at 5 years were determined by the last available value between 4.5 years and 5.5 years after baseline. Any drop-out or loss to-follow-up before the included period were excluded from further analyses. The proportions of patients having each of 5 CVD risk factors at or below target values in RAMP-HT group were compared with those of the usual care group using logistic regression, adjusted for baseline value. For each clinical outcome measures, 5-year cumulative incidences, incidence rates, and corresponding absolute risk reduction in each group were reported. Kaplan-Meier survival curves for event incidences were compared by log-rank test. Multivariable Cox proportional hazards regressions were performed to estimate the association of RAMP-HT with the risk of each event outcome adjusted for baseline characteristics. Proportional hazards assumption was checked by examining plots of the scaled Schoenfeld residuals against time for the covariates. Multicollinearity was assessed by variance inflation factor. All models satisfied the proportional hazards assumption and no multicollinearity existed (variance inflation factor = 1.94). The number needed to treat for each outcome was calculated based on corresponding hazard ratios (HR).^22^ Frequencies of health service use were compared using negative binomial regression adjusted for baseline characteristics and corresponding incidence rate ratios (IRRs) were calculated.

Eight sensitivity analyses were conducted to evaluate robustness of main results. First, to minimize reverse causality, matched patients with less than 1-year follow-up were excluded. Second, one-to-one propensity score matching with multiple imputation without fine stratification weighting was used. Third, all eligible RAMP-HT participants and usual care patients were included in the analysis after multiple imputation without propensity score matching or weightings. Fourth, fine stratification weighting without multiple imputation was performed. Fifth, propensity score matching without multiple imputation was conducted. Sixth, a complete case analysis including only patients with complete data sets was performed. Lastly, additional analyses using data sets with 25 and 50 multiple imputations with propensity score fine stratification weightings were performed.

All outcomes were further compared in subgroups, stratified by gender, age, smoking status, SBP, fasting glucose, LDL-C, eGFR, body mass index (BMI, calculated as weight in kilograms divided by height in meters squared), estimated Framingham 10-year CVD risk, the Charlson Comorbidity Index, and number and classes of antihypertension drugs and lipid-lowering agents prescribed. Fine stratification weightings were carried out for each subgroup analysis. Interactions between the RAMP-HT effect and each group were assessed, significance of interactions were based on Hommel-adjusted *P* values.^23^

All tests were 2-tailed and *P* < .05 was considered statistically significant. Statistical analyses were performed with Stata version 13.0 (StataCorp) from January 2019 to March 2023.

## Results

A total of 212 707 primary care patients with uncomplicated hypertension (108 045 patients in the RAMP-HT group; 104 662 patients in the usual care group) were matched with fine stratification weightings after multiple imputations and included in the analyses. The patient inclusion flow was illustrated in eFigure 2 in Supplement 1. Data completion rates for all baseline covariates ranged from 69.9% to 100% (eTable 2 in Supplement 1). Baseline characteristics of the RAMP-HT and usual care groups before and after multiple imputations before matching (eTable 3 in Supplement 1), and propensity scores distributions between RAMP-HT group and usual care group (eFigure 3 in Supplement 1) were similar.

Table 1 describes the characteristics of RAMP-HT participants (mean [SD] age: 66.3 [12.3] years; 62 277 [57.6%] were female) and usual care patients (mean [SD] age 66.3 [13.5] years; 60 497 [57.8%] were female) at baseline. The SMD in all characteristics were below 0.1, indicating balance between the 2 groups was achieved by weightings. After a median follow-up of 5.4 years, each RAMP-HT participant attended 2.1 risk assessments, 0.3 nurse intervention, and 0.1 specialist consultation on average (eTable 4 in Supplement 1). The means of SBP, diastolic BP, fasting glucose, LDL-C, and BMI measurements of RAMP-HT and usual care groups during 1 to 4 years of follow-up were plotted in eFigure 4 in Supplement 1. At 5-year follow-up, a total of 72 966 RAMP-HT participants (72.9%) had BP levels at or below target (vs 56 103 [66.5%] in the usual care group), and 64 012 RAMP-HT participants (67.3%) had LDL-C levels at or below target (vs 46 048 [61.8%] in the usual care group (Table 2). After adjustments for baseline values, RAMP-HT participants had 36% higher odds (OR, 1.36; 95% CI, 1.33-1.39) of having all 5 CVD risk factors being at or below target values compared to usual care patients. A greater increase in the proportion of patients prescribed statins was observed in the RAMP-HT group (RAMP-HT: from 7.8% to 39.4% vs usual care: from 7.3% to 32.9%) (eTable 5 in Supplement 1).

**Table 1.** Characteristics of RAMP-HT and Usual Care Patients at Baseline After Multiple Imputation and Fine Stratification Weightings

Characteristics	RAMP-HT (n = 108 045)	Usual care (n = 104 662)	SMD^a^
Sex, No. (%)			
Female	62 277 (57.6)	60 497 (57.8)	0.003
Male	45 768 (42.4)	44 165 (42.2)	
Age, mean (SD), year	66.3 (12.3)	66.3 (13.5)	<0.001
Smoking status			
Smoker	8470 (7.8)	8021 (7.7)	0.007
Clinical characteristics, mean (SD)			
SBP, mm Hg	136.6 (16.5)	136.5 (17.8)	0.004
DBP, mm Hg	76.3 (11.4)	76.3 (11.6)	<0.001
Fasting glucose, mmol/L	5.4 (1.2)	5.4 (0.7)	<0.001
LDL-C, mmol/L	3.2 (0.9)	3.2 (0.9)	0.005
Total cholesterol/HDL-C ratio	4.0 (2.2)	4.0 (1.6)	0.001
Triglyceride, mmol/L	1.5 (1.0)	1.5 (1.0)	<0.001
BMI	25.4 (4.1	25.4 (5.2)	<0.001
eGFR, No. (%)			
≥60 mL/min/1.73m^2^	102 906 (95.2)	99 849 (95.4)	0.007
<60 mL/min/1.73m^2^	5139 (4.8)	4813 (4.6)	
Charlson Comorbidity Index	3.1 (1.2)	3.1(1.4)	0.003
No. of anti-hypertensive drugs prescribed, No. (%)			
0-1	62 506 (56.5)	57 173 (56.0)	0.009
≥2	45 539 (43.5)	47 489 (44.0)	
Prescription of ACEI/ARB, No. (%)	21 259 (19.7)	20 253 (19.4)	0.008
Prescription of β-blocker, No. (%)	40 026 (37.0)	38 763 (37.0)	<0.001
Prescription of CCB, No. (%)	76 077 (70.4)	73 571 (70.3)	0.003
Prescription of diuretic, No. (%)	13 521 (12.5)	13 041 (12.5)	0.002
Prescription of other antihypertensive drugs, No. (%)	11 674 (10.8)	11 189 (10.7)	0.004
Prescription of statin, No. (%)	8447 (7.8)	7685 (7.3)	0.018
Prescription of fibrate, No. (%)	1938 (1.8)	1821 (1.7)	0.004
Frequency of service use, mean (SD)^b^			
Overnight hospitalization	0.2 (1.1)	0.2 (0.5)	0.007
Accident and emergency department	0.4 (2.5)	0.4 (1.1)	0.004
Specialist outpatient clinic	1.7 (3.6)	1.7 (3.0)	0.003
General outpatient clinic	5.6 (3.5)	5.6 (2.7)	0.023

Abbreviations: ACEI, angiotensin converting enzyme inhibitor; ARB, angiotensin receptor blocker; BMI, body mass index (calculated as weight in kilograms divided by height in meters squared); CCB, calcium channel blocker; DBP, diastolic blood pressure; eGFR, estimated glomerular filtration rate; HDL-C, high-density lipoprotein cholesterol; LDL-C, low-density lipoprotein cholesterol; RAMP-HT, Risk Assessment and Management Program for Hypertension; SBP, systolic blood pressure; SMD, standardized mean difference.

SI unit conversion factors: To convert glucose to mg/dL, divide by 0.0555; to convert LDL-C to mg/dL, divide by 0.0259; to convert triglyceride to mg/dL, divide by 0.0113.

^a^ SMD <0.2 indicates balance between groups.

^b^ Service use was measured at baseline: from a year before baseline to baseline.

**Table 2.** Proportion of Patients in the RAMP-HT or Usual Care Achieving Clinical Targets at Baseline and 5 Years

Variable	No. (%)		OR (95%) for RAMP-HT group achieving target adjusted for baseline value^a^
	Baseline	At 5 y	
SBP <140 mm Hg and DBP <90mm Hg			
RAMP-HT participants	57 330 (57.2)	72 966 (72.9)	1.37 (1.34-1.40)
Non–RAMP-HT participants	50 178 (59.5)	56 103 (66.5)	NA
OR for RAMP-HT achieving target	0.94 (0.93-0.96)	1.35 (1.32-1.38)	NA
LDL-C (<3.4 mmol/L for Framingham 10-y CVD risk ≤20% or <2.6 mmol/L for Framingham 10-y CVD risk >20%)			
RAMP-HT participants	41 886 (44.0)	64 012 (67.3)	1.31 (1.28-1.33)
Non–RAMP-HT participants	33 215 (44.6)	46 048 (61.8)	NA
OR for RAMP-HT achieving target	0.98 (0.97-1.00)	1.27 (1.25-1.30)	NA
FG <5.6 mmol/L			
RAMP-HT participants	73-968 (68.5)	60-966 (63.1)	1.22 (1.19-1.25)
Non–RAMP-HT participants	70 757 (67.6)	46 170 (58.4)	NA
OR for RAMP-HT achieving target	1.04 (1.02-1.06)	1.22 (1.20-1.24)	NA
BMI <27.5			
RAMP-HT participants	79 566 (73.6)	61 907 (72.9)	1.01 (0.98-1.05)
Non–RAMP-HT participants	76 214 (72.8)	42 451 (71.1)	NA
OR for RAMP-HT achieving target	1.04 (1.02-1.07)	1.10 (1.07-1.12)	NA
Nonsmokers			
RAMP-HT participants	99 575 (92.2)	101 109 (93.6)	1.16 (1.10-1.24)
Non–RAMP-HT participants	96 641 (92.3)	96 687 (93.4)	NA
OR for RAMP-HT achieving target	0.98 (0.94-1.01)	1.04 (1.00-1.08)	NA
Achieved all 5 targets			
RAMP-HT participants	19 132 (17.7)	47 420 (58.7)	1.36 (1.33-1.39)
Non–RAMP-HT participants	19 252 (18.4)	28 143 (51.8)	NA
OR for RAMP-HT achieving target	0.95 (0.93-0.98)	1.32 (1.29-1.35)	NA

Abbreviations: BMI, body mass index (calculated as weight in kilograms divided by height in meters squared); CVD, cardiovascular disease; DBP, diastolic blood pressure; FG, fasting glucose; LDL-C, low-density lipoprotein cholesterol; NA, not applicable; OR, odds ratio, RAMP-HT, Risk Assessment and Management Program for Hypertension; SBP, systolic blood pressure.

SI unit conversion factors: To convert glucose to mg/dL, divide by 0.0555; to convert LDL-C to mg/dL, divide by 0.0259.

^a^ OR were adjusted for the corresponding target of clinical outcome at baseline.

The 5-year cumulative incidence of the primary composite outcome was 11.8% (n = 12 784) in RAMP-HT group and 26.3% (n = 27 514) in the usual care group (Table 3), corresponding to an absolute risk reduction of 14.5% for RAMP-HT participants. RAMP-HT participants had an absolute risk reduction of 8.0% in CVD, 1.6% in ESKD, and 10.0% in all-cause mortality. Between-group differences in cumulative hazards for adverse outcomes were found as early as 1 year after baseline (Figure), and continued at least up to the fifth year. After adjusting for all baseline covariates, RAMP-HT participants had 42% lower risk of composite outcome (HR, 0.58; 95% CI, 0.57-0.59), 38% lower risk of CVD (HR, 0.62; 95% CI, 0.61-0.64), 46% lower risk of ESKD (HR, 0.54; 95% CI, 0.50-0.59), 17% lower risk of diabetes (HR, 0.83; 95% CI, 0.80-0.85), and 48% lower risk of all-cause mortality (HR, 0.52; 95% CI, 0.50-0.54) compared with the usual care group. The number needed to treat to prevent 1 event was 11 for all composite events, 16 for CVD, 106 for ESKD, 41 for diabetes, and 17 for all-cause mortality. Consistently, there were reductions in overnight hospitalization (IRR, 0.60), accident and emergency department attendances (IRR, 0.70), and specialist outpatient clinic attendances (IRR, 0.87), whereas GOPC attendances were 6% higher (IRR, 1.06) in the RAMP-HT group (Table 4). Results from the 8 sensitivity analyses were similar to the main analyses (eTables 6 and 7 in Supplement 1).

**Table 3.** Outcome Events at 5 Years in RAMP-HT Participants and Usual Care Patients

Event	RAMP-HT participants (n = 108 045)		Usual care patients (n = 104 662)		ARR, %	HR^a^	*P* value	NNT
	Cases with event, No. (%)	Incidence rate (cases/100 person years) (95% CI)	Cases with event, No. (%)	Incidence rate (cases/100 person-years) (95% CI)				
All outcome event	12 784 (11.8)	3.1 (3.0-3.1)	27 514 (26.3)	5.4 (5.3-5.5)	14.5	0.58 (0.57-0.59)	<.001	11 (10-11)
CVD	9167 (8.5)	2.1 (2.1-2.2)	17 261 (16.5)	3.4 (3.3-3.4)	8.0	0.62 (0.61-0.64)	<.001	16 (15-16)
CHD	3964 (3.7)	0.9 (0.8-0.9)	6607 (6.3)	1.3 (1.2-1.3)	2.6	0.66 (0.63-0.69)	<.001	47 (44-51)
Heart failure	1799 (1.7)	0.4 (0.4-0.5)	5094 (4.9)	0.9 (0.9-0.9)	3.2	0.54 (0.51-0.58)	<.001	49 (46-52)
Stroke	4378 (4.1)	1.0 (1.0-1.0)	8467 (8.1)	1.6 (1.5-1.6)	4.0	0.64 (0.61-0.66)	<.001	36 (33-38)
ESKD	808 (0.7)	0.2 (0.2-0.2)	2409 (2.3)	0.4 (0.4-0.4)	1.6	0.54 (0.50-0.59)	<.001	106 (97-120)
Diabetes	10235 (9.5	2.4 (2.4-2.5)	14 724 (14.1)	3.1 (3.1-3.2)	4.6	0.83 (0.80-0.85)	<.001	41 (36-48)
All-cause mortality	4833 (4.5)	1.2 (1.2-1.3)	15 144 (14.5)	2.8 (2.7-2.8)	10.0	0.52 (0.50-0.54)	<.001	17 (16-18)
CVD mortality	1532 (1.4)	0.4 (0.4-0.4)	576 (5.3)	1.0 (1.0-1.1)	3.9	0.51 (0.48-0.54)	<.001	43 (40-46)
Non-CVD mortality	3301 (3.1)	0.8 (0.8-0.8)	9568 (9.1)	1.8 (1.7-1.8)	6.0	0.54 (0.51-0.56)	<.001	45 (43-48)

Abbreviations: ARR, absolute risk reduction; CHD, coronary heart disease; CVD, cardiovascular disease; ESKD, end-stage kidney disease; HR, hazard ratio; NNT, number needed to treat; RAMP-HT, Risk Assessment and Management Program for Hypertension.

^a^ Hazard ratios were adjusted by gender, age, smoking status, systolic blood pressure, diastolic blood pressure, fasting glucose, low-density lipoprotein cholesterol, TC/HDL-C ratio, triglyceride, body mass index, estimated glomerular filtration rate, Charlson Comorbidity Index and the usages of angiotensin converting enzyme inhibitor/angiotensin receptor blocker, β-blocker, calcium channel blocker, diuretic, other antihypertensive drugs, statin and fibrate at baseline.

**Figure.**
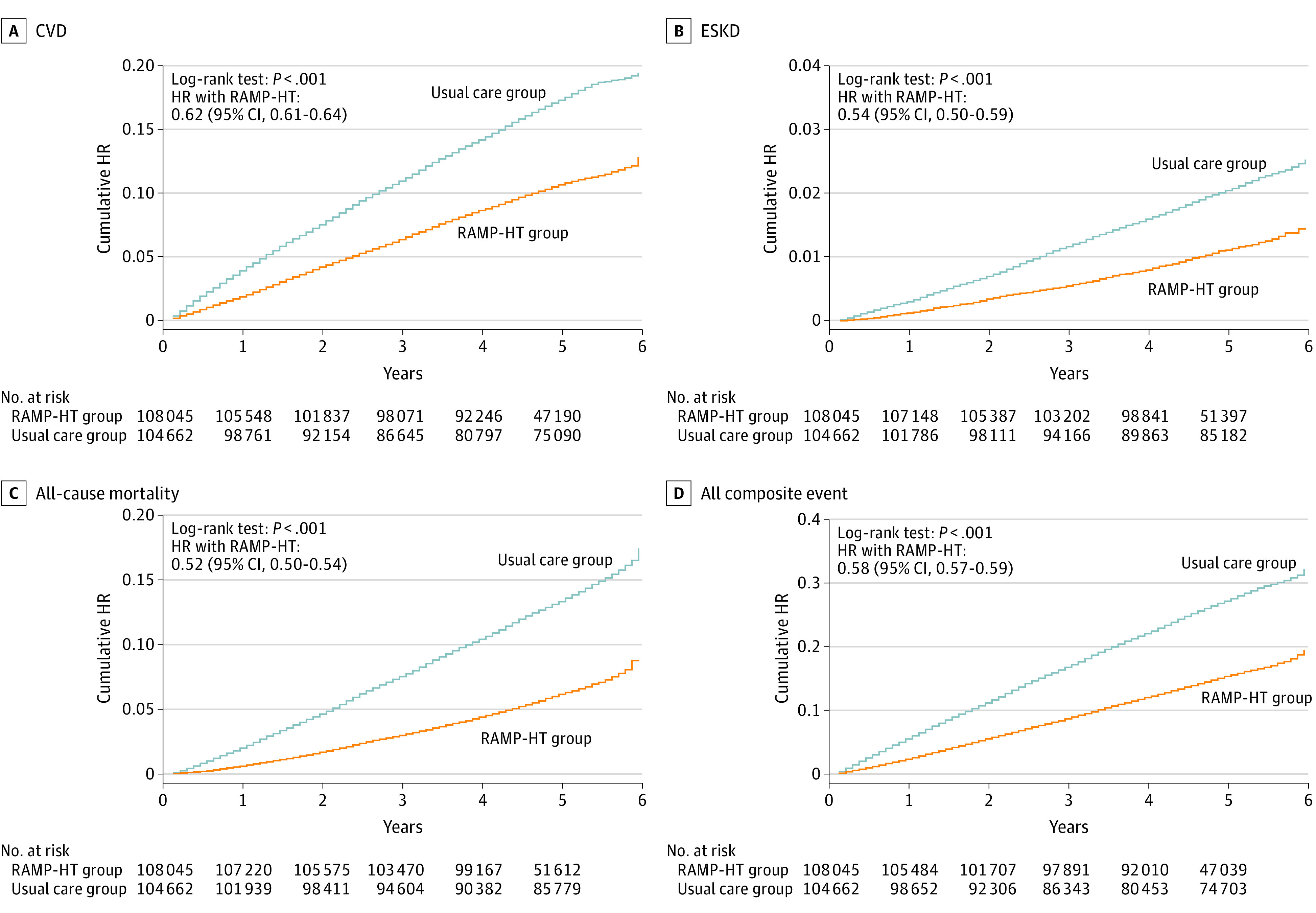
Cumulative Hazards for Cardiovascular Disease, End-Stage Kidney Disease, All-Cause Mortality, and All Composite Events of Primary Outcome Between RAMP-HT and Usual Care Groups HR indicates hazard ratio; RAMP-HT, Risk Assessment and Management Program for Hypertension.

**Table 4.** Public Health Service Use of RAMP-HT Participants and Usual Care Patients at 5 Years

Service	RAMP-HT participants (n = 108 045)						Usual care patients (n = 104 662)						IRR (95% CI)^a^	P value
	Mean (SD)	Median	IQR	Range	Total No. of events	Event rate (cases/100 person years)	Mean (SD)	Median	IQR	Range	Total No. of events	Event rate (cases/100 person years)		
Overnight hospitalization	1.11 (0.01)	0	1	0-69	119 670	23.4	1.90 (0.01)	1	3	0-105	199 951	36.7	0.60 (0.59-0.61)	<.001
AED attendance	1.88 (0.02)	1	2	0-200	203 769	39.9	2.80 (0.02)	2	4	0-1073	293 703	53.9	0.70 (0.69-0.71)	<.001
SOPC attendance	9.05 (0.04)	4	12	0-126	979 227	191.5	10.86 (0.04)	6	16	0-254	1 141 245	209.6	0.87 (0.86-0.88)	<.001
GOPC attendance	21.03 (0.04)	20	8	0-422	2 276 471	445.2	22.27 (0.04)	22	15	0-1381	2 339 024	429.5	1.06 (1.06-1.06)	<.001

Abbreviations: AED, accident and emergency department; GOPC, general outpatient clinics; IRR, incidence rate ratio; RAMP-HT, Risk Assessment and Management Program for Hypertension; SOPC, specialist outpatient clinics.

^a^ All incidence rate ratios were adjusted by gender, age, smoking status, systolic blood pressure, diastolic blood pressure, fasting glucose, LDL-C, TC/HDL-C ratio, triglyceride, BMI, eGFR, Charlson Comorbidity Index and the usages of ACEI/ARB, β-blocker, CCB, diuretic, other antihypertensive drugs, statin and fibrate, and the corresponding number of service uses at baseline.

Further analyses showed greater risk reduction in the incidences of adverse events and less hospital-based service use (eFigure 5 in Supplement 1) in RAMP-HT participants regardless of subgroups. Participants aged 80 years or older and those with fasting glucose of at least 6.1 mmol/L exhibited a smaller reduction in risk of adverse events than other groups.

## Discussion

Implementation of the RAMP-HT added onto to public usual care was associated with significant reductions in the absolute risks of incident CVD (−8.0%), ESKD (−1.6%), and all-cause mortality (−10.0%) among primary care patients with hypertension compared with usual care alone. A corresponding decrease in hospital-based service use was observed.

Reductions in CVD and mortality associated with RAMP-HT might partly be attributed to the synergistic effects of BP and LDL-C control. RAMP-HT participants had a higher likelihood of achieving BP and LDL-C control compared with usual care patients after 5 years, where a greater proportion of RAMP-HT participants were prescribed statins, amid increased statins use in both groups since introduction of the drug in GOPC formulary shortly before launch of the program. RAMP-HT adopted a treat-to-target approach for total CVD risk management, thus explaining for the relatively small absolute differences in BP and LDL-C between groups. However, absolute risk reductions (1.6% to 14.5%) for adverse outcomes were substantial. These findings were comparable to the SPLINT trial, where a specialist nurse-led hypertension/dyslipidaemia clinic resulted in significantly lower mortality (OR, 0.55; 95% CI, 0.32-0.92) after 1 year despite similar BP reduction between groups.^24^ While the magnitude of risk reduction in this study was considerably greater than many large-scale drug trials,^25,26,27^ it was consistent with findings from the HOPE-3 trial^28^ and ASCOT-LLA trial^29^ where use of statins in addition to BP-lowering medications reduced risk of CVD events by 40% to 50% in individuals with hypertension. A meta-analysis of 61 prospective studies further demonstrated an approximately additive effect of BP and total cholesterol reduction on lowering ischemic heart disease mortality,^30^ reinforcing the importance of total CVD risk reduction among patients with hypertension to prevent adverse outcomes.

The add-on nurse-led risk assessment and intervention sessions contributed further through patient empowerment on self-care, including self-BP monitoring, smoking cessation, dietary modifications, physical activity, medication adherence and help-seeking behavior.^11,31^ The potential contribution of these interventions might be proxied from the greater likelihood for RAMP-HT participants to be nonsmokers, and have all 5 CVD risks being at or below target values after 5 years. The strategic risk-stratified management also allowed for better care coordination and resource allocation. High-risk RAMP-HT participants with active health problems were channeled to attend early GOPC follow-up for review and management, hereby explaining for their higher GOPC attendance but reduced use of other hospital-based health care services.

From a post hoc analysis of the landmark Steno-2 trial in patients with diabetes, multifactorial intervention demonstrated modest improvements in disease parameters but significant relative risk reduction in CVD (59%) and all-cause mortality (46%) after a mean follow-up of 13 years.^32^ Similarly, the observed reductions in CVD and mortality in this study might represent a function of change in practices on multiple levels beyond disease parameter control. In addition to overcoming clinicians’ treatment inertia through clinician training and use of action reminder prompts, and empowering patients’ self-care capacity through extra contact time with nurses and the allied health care team, task-shifting also allowed more time for physicians to promptly recognize and manage other complex or urgent issues during the time-constrained consultation, which might contribute to the lower incidence of non-CVD mortality in RAMP-HT participants.

Strengths of this study included the inclusion of a large representative cohort of primary care patients with hypertension over the long-term. Our observations reflected how such a model of care benefitted all groups of patients with different levels of literacy, motivation, and baseline health. Several sophisticated analytical techniques including multiple imputations, fine stratification weightings and adjustments of inclusion criteria were used to minimize imbalances in baseline characteristics, and reduce the contributions of confounding between groups. The use of weighting in addition to propensity-score matching allowed for the maximum use of data and participants contributing differing amounts of information depending on their weights, which offered a more efficient and precise estimation of treatment effects.^33,34^ The comprehensive list of confounding variables and subgroups analyses reinforced the reliability and robustness of the results. Furthermore, consistent results obtained in the series of sensitivity analyses suggested that outcomes of RAMP-HT could be generalized regardless of the statistical approaches used. Comparison of RAMP-HT with usual care was comprehensive, including surrogate markers, actual CVD events and service use. All information obtained from the administrative database was widely used in all clinics and hospitals and were systematically managed by the Hospital Authority, thus ensuring data accuracy and reliability.

### Limitations

There were also several limitations to this study. First, the prospective cohort design does not prove cause and effect. Despite the array of statistical approaches used to minimize potential biases, these biases could not be eliminated entirely. Propensity-score matching might aid in removing confounding effects from measured baseline covariates, but residual confounding from unmeasured covariates still existed. Common to studies based on electronic medical records, socioeconomic variables and behavioral factors such as motivation, diet and physical activity were not captured in this study. RAMP-HT participants might inherently be more motivated and adherent to interventions, contributing to possible selection bias, and the potential for results to bias away from the null. Second, all GOPC physicians have been trained to deliver standardized hypertension care according to the latest Hong Kong reference framework for hypertension in primary care, which recommendations were similar to the RAMP-HT protocol. Consequently, remarkable improvement in CVD risks was observed also in the usual care group, possibly leading to contamination bias. Third, no validation was performed as part of this study to assess the accuracy and completeness of outcome events coding. Nevertheless, a previous study showed that only up 5.5% of records were miscoded or lacked coding for diabetes diagnosis in the HA-CMS.^35^ Fourth, event outcomes and service use measured in this study were limited to those available within the public health care system. However, patients with chronic diseases and serious complications were mostly treated in the heavily subsidized public system; hence our data should have captured the majority of CVD and ESKD outcomes. Additionally, individual contribution of different elements of RAMP-HT to its overall impact was not assessed. The RAMP-HT was implemented in an integrated primary care system fully funded by the government. Direct translation of these findings may be limited to contexts with similar incentives, financial investment, and infrastructure support.

## Conclusions

The RAMP-HT—a protocol-driven, team-based, multicomponent intervention integrated into usual care—was associated with significant reduction of hypertension-related complications and all-cause mortality among patients with hypertension in the public primary care setting after 5 years. The corresponding reduction in hospital-based service use highlighted its potential to alleviate public health care burden. Further study should be conducted to determine the cost-effectiveness and long-term sustainability of the program.

## Notes

Supplement 1. **eMethods 1.** Setting of RAMP-HT and Usual Care **eMethods 2.** Baseline Covariates **eFigure 1.** Risk Assessment & Management Program – Hypertension (RAMP-HT) Workflow **eFigure 2.** Patient Inclusion Flow Chart **eFigure 3.** Propensity Scores Distributions of RAMP-HT Group and Usual Care Group **eFigure 4.** Means of Systolic Blood Pressure (SBP), Diastolic Blood Pressure (DBP), Fasting Glucose (FG), Low Density Lipoprotein – Cholesterol (LDL-C) and Body Mass Index (BMI) of RAMP-HT Group and Usual Care Group **eFigure 5.** Adjusted Hazard Ratios (HRs) of RAMP-HT Participants Over Usual Care Patients Associated With the Incidences of Cardiovascular Diseases (CVD), End-Stage Renal Disease (ESRD), All-Cause Mortality and All Composite Event in Selected Subgroups by Multivariable Cox Proportional Hazards Regressions **eFigure 6.** Adjusted Incident Rate Ratios (IRRs) of RAMP-HT Participants Over Usual Care Patients Associated With the Number of Hospitalization, Accident and Emergency Department (AED) Attendance, Special Outpatient Clinic (SOPC) and General Outpatient Clinic (GOPC) Attendance in Selected Subgroups by Negative Binomial Regressions **eTable 1.** Definition of the Event Outcome Measures **eTable 2.** Data Completion Rates of Matched RAMP-HT Participants and Usual Care Patients at Baseline and 5-Year Follow-up **eTable 3.** Baseline Characteristics Between RAMP-HT Participants and Usual Care Patients Before and After Multiple Imputation Without Fine Stratification Weightings **eTable 4.** Frequency of RAMP-HT Service Attendances Among RAMP-HT Participants **eTable 5.** Patients’ Characteristics Between RAMP-HT Participants and Usual Care Patients at 5-Year Follow-up After Fine Stratification Weightings **eTable 6.** Sensitivity Analyses on Comparisons of All Outcome Events, Diabetes Mellitus, Cardiovascular Disease, End Stage Renal Disease and All-Cause Mortality Between RAMP-HT Participants and Usual Care Patients **eTable 7.** Sensitivity Analyses on Comparisons of Service Use Between RAMP-HT Participants and Usual Care Patients

Supplement 2.
Data Sharing Statement
