# Supplementary material for: Assessment of Hypertension Complications and Health Service Use 5 Years After Implementation of a Multicomponent Intervention
Source: JAMA Netw Open. 2023 May 24;6(5):e2315064. doi: 10.1001/jamanetworkopen.2023.15064 (PMC10209746; doi:10.1001/jamanetworkopen.2023.15064)
Supplement: Supplement 1. — eMethods 1. Setting of RAMP-HT and Usual Care eMethods 2. Baseline Covariates eFigure 1. Risk Assessment & Management Program – Hypertension (RAMP-HT) Workflow eFigure 2. Patient Inclusion Flow Chart eFigure 3. Propensity Scores Distributions of RAMP-HT Group and Usual Care Group eFigure 4. Means of Systolic Blood Pressure (SBP), Diastolic Blood Pressure (DBP), Fasting Glucose (FG), Low Density Lipoprotein – Cholesterol (LDL-C) and Body Mass Index (BMI) of RAMP-HT Group and Usual Care Group eFigure 5. Adjusted Hazard Ratios (HRs) of RAMP-HT Participants Over Usual Care Patients Associated With the Incidences of Cardiovascular Diseases (CVD), End-Stage Renal Disease (ESRD), All-Cause Mortality and All Composite Event in Selected Subgroups by Multivariable Cox Proportional Hazards Regressions eFigure 6. Adjusted Incident Rate Ratios (IRRs) of RAMP-HT Participants Over Usual Care Patients Associated With the Number of Hospitalization, Accident and Emergency Department (AED) Attendance, Special Outpatient Clinic (SOPC) and General Outpatient Clinic (GOPC) Attendance in Selected Subgroups by Negative Binomial Regressions eTable 1. Definition of the Event Outcome Measures eTable 2. Data Completion Rates of Matched RAMP-HT Participants and Usual Care Patients at Baseline and 5-Year Follow-up eTable 3. Baseline Characteristics Between RAMP-HT Participants and Usual Care Patients Before and After Multiple Imputation Without Fine Stratification Weightings eTable 4. Frequency of RAMP-HT Service Attendances Among RAMP-HT Participants eTable 5. Patients’ Characteristics Between RAMP-HT Participants and Usual Care Patients at 5-Year Follow-up After Fine Stratification Weightings eTable 6. Sensitivity Analyses on Comparisons of All Outcome Events, Diabetes Mellitus, Cardiovascular Disease, End Stage Renal Disease and All-Cause Mortality Between RAMP-HT Participants and Usual Care Patients eTable 7. Sensitivity Analyses on Comparisons of Service Use Between RAMP-HT Participants and [file jamanetwopen-e2315064-s001.pdf]

Supplemental Online Content

Yu EYT, Wan EYF, Mak IL, et al. Assessment of hypertension complications and health service use 5 years after implementation of a multicomponent intervention. *JAMA Netw Open.* 2023;6(5):e2315064. doi:10.1001/jamanetworkopen.2023.15064

eMethods 1. Setting of RAMP-HT and Usual Care
eMethods 2. Baseline Covariates
eFigure 1. Risk Assessment & Management Program – Hypertension (RAMP-HT) Workflow
eFigure 2. Patient Inclusion Flow Chart
eFigure 3. Propensity Scores Distributions of RAMP-HT Group and Usual Care Group
eFigure 4. Means of Systolic Blood Pressure (SBP), Diastolic Blood Pressure (DBP), Fasting Glucose (FG), Low Density Lipoprotein – Cholesterol (LDL-C) and Body Mass Index (BMI) of RAMP-HT Group and Usual Care Group
eFigure 5. Adjusted Hazard Ratios (HRs) of RAMP-HT Participants Over Usual Care Patients Associated With the Incidences of Cardiovascular Diseases (CVD), End-Stage Renal Disease (ESRD), All-Cause Mortality and All Composite Event in Selected Subgroups by Multivariable Cox Proportional Hazards Regressions
eFigure 6. Adjusted Incident Rate Ratios (IRRs) of RAMP-HT Participants Over Usual Care Patients Associated With the Number of Hospitalization, Accident and Emergency Department (AED) Attendance, Special Outpatient Clinic (SOPC) and General Outpatient Clinic (GOPC) Attendance in Selected Subgroups by Negative Binomial Regressions
eTable 1. Definition of the Event Outcome Measures
eTable 2. Data Completion Rates of Matched RAMP-HT Participants and Usual Care Patients at Baseline and 5-Year Follow-up
eTable 3. Baseline Characteristics Between RAMP-HT Participants and Usual Care Patients Before and After Multiple Imputation Without Fine Stratification Weightings
eTable 4. Frequency of RAMP-HT Service Attendances Among RAMP-HT Participants
eTable 5. Patients' Characteristics Between RAMP-HT Participants and Usual Care Patients at 5-Year Follow-up After Fine Stratification Weightings
eTable 6. Sensitivity Analyses on Comparisons of All Outcome Events, Diabetes Mellitus, Cardiovascular Disease, End Stage Renal Disease and All-Cause Mortality Between RAMP-HT Participants and Usual Care Patients
eTable 7. Sensitivity Analyses on Comparisons of Service Use Between RAMP-HT Participants and Usual Care Patients

This supplemental material has been provided by the authors to give readers additional information about their work.

eMethods 1. Setting of RAMP-HT and Usual Care

Risk Assessment and Management Program for Hypertension (RAMP-HT)

The RAMP-HT introduced three specific services to augment the time-constrained usual care: risk assessment, nurse intervention and specialist consultation.

All participants would undergo a comprehensive, protocol-guided risk assessment conducted by a trained nurse, which involves standardized cardiovascular disease (CVD) risk assessment (including smoking habit, blood pressure (BP) level, lipid profile, glycaemic status and obesity), hypertensive complication screening (including the presence of left ventricular hypertrophy, arrhythmia and/or ischaemic heart disease by electrocardiogram, cerebrovascular disease from medical history, peripheral vascular disease by vascular doppler assessment, chronic kidney disease and proteinuria by laboratory testing), and detailed assessment on self-care (including medication adherence, smoking, diet and exercise habits, and self-BP monitoring practice). Each participant would then be stratified as having low, medium or high risk according to the 10-year predicted CVD risk calculated using the Joint British Societies' (JBS2) risk calculator.[1] Based on individual risk level, presence of other CVD risk factors and participants' preference, the nurse – serving as care-manager, would empower the participants on self-care (e.g. lifestyle and self-BP monitoring), prepare a care plan and coordinate follow-up interventions by a multi-disciplinary team for the participants according to protocol. To enhance communication and facilitate clinical decision-making of team members, the care-manager record would record each participant's CVD risk profile and care plan on an electronic record platform with an action reminder system, which is accessible to all doctors and nurses from the Hospital Authority clinical management system across all 73 General Out-Patient Clinics (GOPC). The risk assessment might be repeated every 12-30 months depending on the participant's risk profile.

Participants with adherence issues or specific risk factors would be referred for nurse interventions; participants with resistant hypertension (i.e. SBP ≥ 160/100 mm Hg despite taking at least 3 anti-hypertensive medications) would be referred for additional specialist consultations. High risk participants who were found to have suboptimal blood pressure, lipid or glycaemic control, abnormal findings from investigations, problems with drug compliance, and new symptoms suggestive of hypertension-related complications or adverse effects from treatment would be channelled to an early GOPC follow-up. Meanwhile, all participants would continue to receive care from their usual primary care doctors every 8-16 weeks at GOPCs, who would receive an electronic report on the participants' CVD risk control and additional intervention received, and an action reminder to facilitate medication titration. The usual care doctors might also provide care for other co-morbidities and new health problems during the scheduled follow-ups.

Two cycles of evaluation on the quality of care of the RAMP-HT were conducted in 2013 and 2015, which confirmed satisfactory adherence to the RAMP-HT protocol.[2]

To implement the RAMP-HT as an add-on intervention to usual care, RAMP-HT services were gradually set up at 59 out of 73 GOPCs in Hong Kong between October 2011 to September 2013 to serve patients residing in different geographic clusters. All patients aged 18 years or above who had hypertension and were receiving management in any of the 73 GOPCs, but no pre-existing diabetes mellitus (DM), CVD or ESRD were eligible to participate in the RAMP-HT, while

© 2023 Yu EYT et al. *JAMA Network Open.*

patients with concomitant DM were channeled for enrollment in the parallel Risk Assessment and Management Program for Diabetes Mellitus (RAMP-DM) instead.[3] Patients were recruited by their attending usual care doctors or nurses to voluntarily enroll in the RAMP-HT during their scheduled follow-up visits. Patients under care of a GOPC without RAMP-HT service would be invited to enroll in RAMP-HT services at a nearest RAMP-HT clinic within the same geographic district, while continue to receive usual follow-up care at their home clinic with the support of the electronic action reminder system.

Although the RAMP-HT is a territory-wide program intended for all patients with hypertension managed in GOPCs, roll-out of the program could only be performed in stages due to the large and ever-increasing number of patients, limited resources and manpower. Therefore, this represented an opportunity to examine the outcomes of RAMP-HT compared to usual care prior to its complete implementation in the public primary care setting.

Usual care

Patients with hypertension are offered a scheduled follow-up consultation with a doctor at a GOPC every 8 to 16 weeks. All GOPC doctors have been trained to deliver standardized hypertension care according to the Hong Kong reference framework for hypertension in primary care.[4] During a typical 5-minutes consultation, the attending doctor may review the patient's BP and control of other risk factors, titrate medication, advise on lifestyle, arrange assessment or refer to allied health professionals as appropriate. Based on clinical judgement, the doctor may also focus on managing the patient's other health problems not directly related to hypertension care during the follow-up appointment. The patients are not required to register with a specific clinic or a regular doctor.

Availability of medications (including statin and angiotensin receptor blockers introduced to the GOPC formulary in 2010), access to all laboratory tests, investigations and allied health professional services in the public primary care clinics and community centers are the same for RAMP-HT participants and usual care patients.

References

1. JBS 2: Joint British Societies' guidelines on prevention of cardiovascular disease in clinical practice. *Heart*. Dec 2005;91 Suppl 5(Suppl 5):v1-52. doi:10.1136/hrt.2005.079988
2. Yu EY, Wan EY, Chan KH, et al. Evaluation of the quality of care of a multi-disciplinary Risk Factor Assessment and Management Programme for Hypertension (RAMP-HT). *BMC Fam Pract*. Jun 19 2015;16(1):71. doi:10.1186/s12875-015-0291-0
3. Fung CS, Chin WY, Dai DS, et al. Evaluation of the quality of care of a multi-disciplinary risk factor assessment and management programme (RAMP) for diabetic patients. *BMC family practice*. 2012;13(1):1-9.
4. Department of Health HS. Hong Kong Reference Framework for Hypertension Care for Adults in Primary Care Settings. Department of Health, HK SAR; 2018.

© 2023 Yu EYT et al. *JAMA Network Open.*

eMethods 2. Baseline Covariates

Baseline covariates included socio-demographics, medical history, and anthropometric measurements and laboratory investigation results that were routinely collected at GOPC follow-up and extracted from the electronic health records from the Hospital Authority Clinical Management System.

Socio-demographics included gender, age and smoking status.

Medical history included the Charlson Comorbidity Index, prescriptions of anti-hypertensive medications including angiotensin converting enzyme inhibitor/angiotensin receptor blocker (ACEI/ARB), β-blocker, calcium channel blocker (CCB), diuretic or other anti-hypertensive medications, and lipid-lowering agents.

Anthropometric measurements included body weight, height, and SBP and diastolic BP (DBP) measured using semi-automated blood pressure measurement devices.

Laboratory results consisted of a full lipid profile (low-density lipoprotein-cholesterol (LDL-C), total cholesterol, high-density lipoprotein-cholesterol (HDL-C) and triglycerides), fasting glucose (FG) levels, and estimated glomerular filtration rate (eGFR) calculated with the abbreviated Modification of Diet in Renal Disease Study formula recalibrated for Chinese.[1] All laboratory assays were performed in laboratories accredited by the College of American Pathologists, the Hong Kong Accreditation Service or the National Association of Testing Authorities, Australia.

References

1. Ma YC, Zuo L, Chen JH, et al. Modified glomerular filtration rate estimating equation for Chinese patients with chronic kidney disease. *J Am Soc Nephrol*. Oct 2006;17(10):2937-44. doi:10.1681/ASN.2006040368

© 2023 Yu EYT et al. *JAMA Network Open.*

eFigure 1. Risk Assessment & Management Program – Hypertension (RAMP-HT) workflow

**Risk Assessment by care-manager nurse**	
**Drug Adherence and Lifestyle**	- Adherence to medication by pill-counting and prescription review<br>- Smoking habit, alcohol consumption<br>- Diet, exercise<br>- Self BP-monitoring
**Physical Examination**	- Blood pressure and pulse<br>- Body weight, body height, BMI and waist circumference<br>- Foot pulse (by vascular Doppler)
**Laboratory Tests**	- Blood tests for full lipid profile, fasting glucose level, and renal function<br>- Urine for protein<br>- Electrocardiogram

↓

**Risk Stratification based on 10-years' Cardiovascular Disease Risk Calculation using the Joint British Societies (JBS2) risk calculator**		
**Low Risk (<10%)**	**Medium Risk (10-20%)**	**High Risk (>20% or with TOD)**

↓

Patient Education

- Explanation of risk level
- HT knowledge, self-care practices, Lifestyle advices
- Coordination of multi-disciplinary risk-guided management

↓

Risk-guided Multi-disciplinary Interventions

**Blood pressure**	**Low Risk (<10%)**	**Medium Risk (10-20%)**	**High Risk (>20% or with TOD)**
140/90 – 160/100 mm Hg	GOPC Doctor for drug titration	GOPC Doctor for drug titration	GOPC Doctor for drug titration + add statins if LDL-C suboptimal
≥ 160/100 mm Hg	GOPC Doctor for drug titration + **RAMP-HT Nurse Intervention**		
≥ 160/100 mm Hg + On ≥ 3 kinds of anti-HT drugs	**Specialist Consultation** for further assessment and management + RAMP-HT Nurse Intervention		

Other Risk-guided Interventions:

Patient Empowerment Program – offer to all HT patients who are willing to attend
Smoking Counselling and Cessation Program/Centre – smokers intending to quit
Dietitian – (1) BMI ≥ 27.5kg/m^2 for weight reduction and (2) special dietary needs and (3) poor dietary control despites nurse intervention offered and (4) pre-diabetes
Physiotherapist – BMI ≥ 27.5kg/m^2 intending to join weight reduction program
Integrated Mental Health Program by occupational therapist – psychological problems

© 2023 Yu EYT et al. *JAMA Network Open.*

eFigure 2. Patient inclusion flow chart

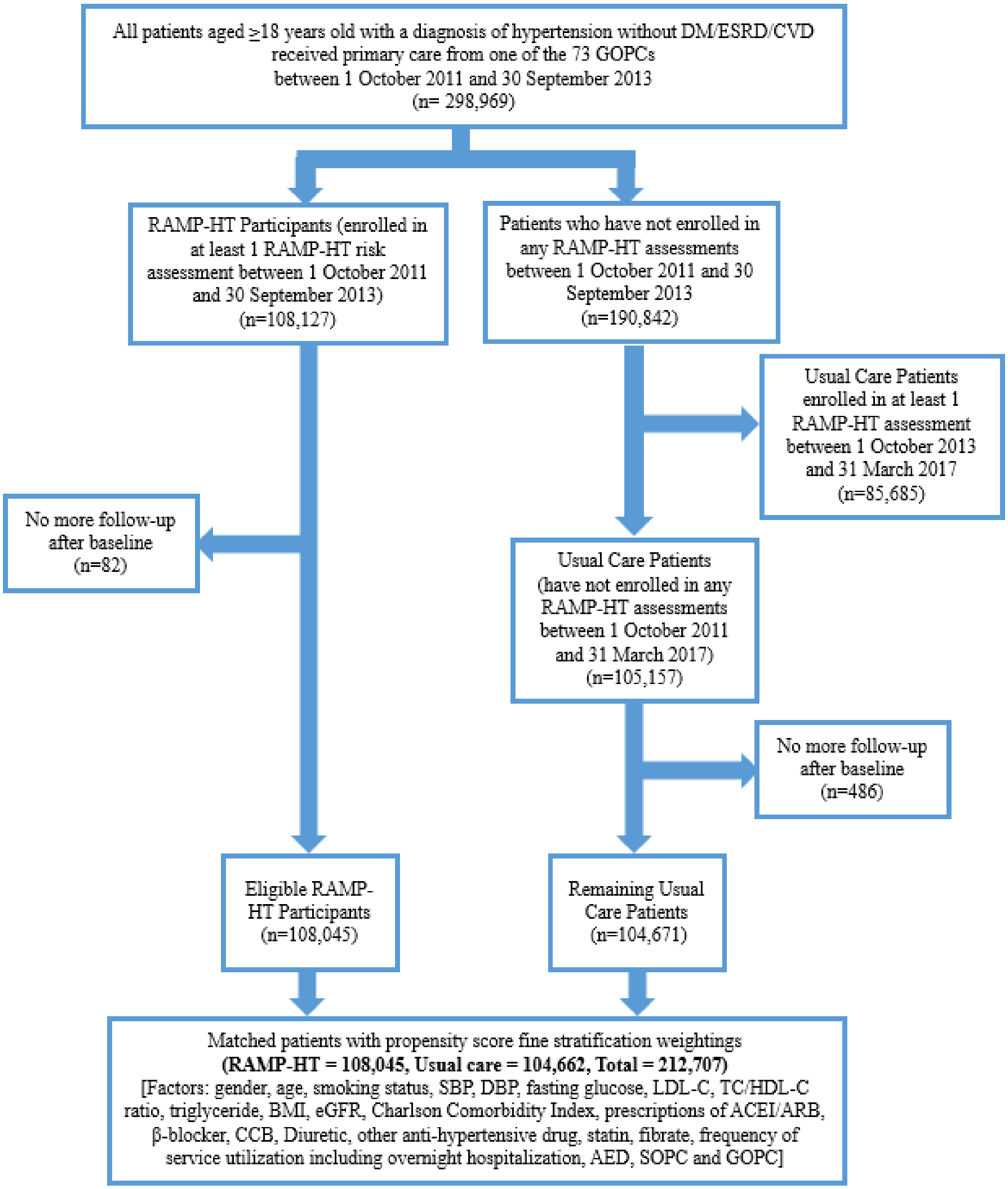

(Abbreviations: ACEI = angiotensin-converting enzyme inhibitors; ARB = angiotensin receptor blockers; AED = accident and emergency department; BMI = body mass index; CCB = calcium channel blockers; CVD = cardiovascular disease; DBP = diastolic blood pressure; DM=Diabetes Mellitus; eGFR = estimated glomerular filtration rate; ESRD = end-stage renal disease; GOPC = general out-patient clinic; HDL-C = high-density lipoprotein-cholesterol; LDL-C = low-density lipoprotein-cholesterol; RAMP-HT = Risk Assessment and Management Program – Hypertension; SBP = systolic blood pressure; SOPC = specialist out-patient clinic; TC = total cholesterol)

© 2023 Yu EYT et al. *JAMA Network Open.*

eFigure 3. Propensity scores distributions of RAMP-HT group and usual care group

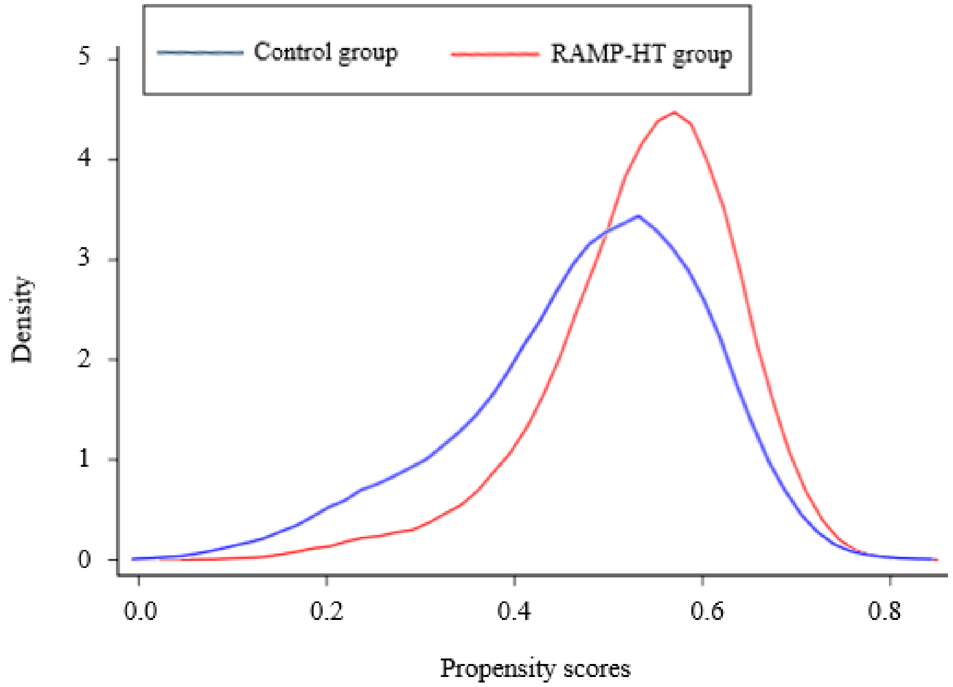

RAMP-HT = Risk Assessment and Management Program – Hypertension

© 2023 Yu EYT et al. *JAMA Network Open.*

eFigure 4. Means of Systolic Blood Pressure, Diastolic Blood Pressure, Fasting Glucose, Low Density Lipoprotein – Cholesterol and Body Mass Index of RAMP-HT group and usual care group

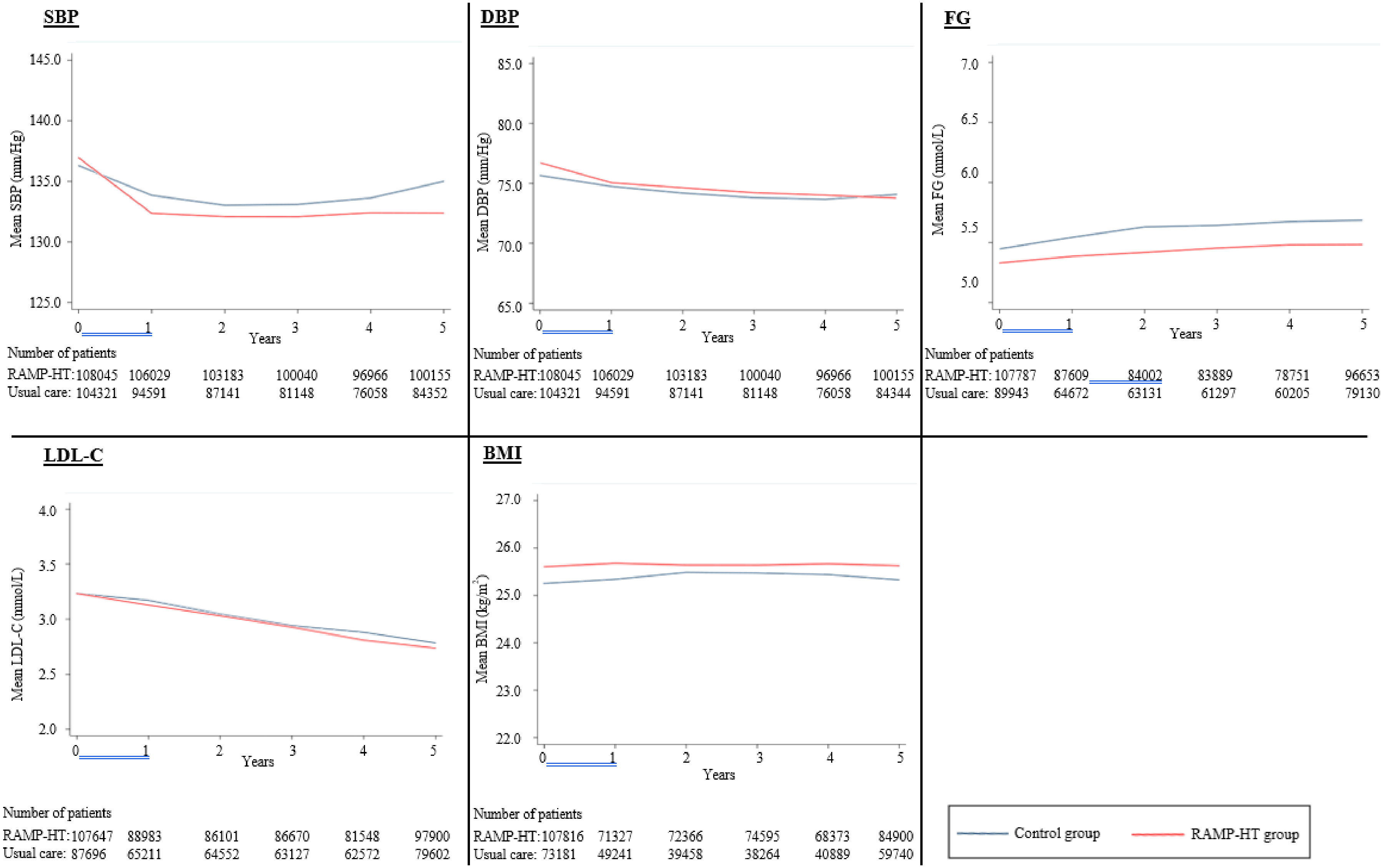

RAMP-HT = Risk Assessment and Management Program – Hypertension; SBP = Systolic Blood Pressure; DBP = Diastolic Blood Pressure; FG = Fasting Glucose; LDL-C = Low Density Lipoprotein – Cholesterol; BMI = Body Mass Index

© 2023 Yu EYT et al. *JAMA Network Open.*

eFigure 5. Adjusted hazard ratios (HRs) of RAMP-HT participants over usual care patients associated with the incidences of cardiovascular diseases (CVD), end-stage renal disease (ESRD), all-cause mortality and all composite event in selected subgroups by multivariable Cox proportional hazards regressions. HR were adjusted for all covariates at baseline.

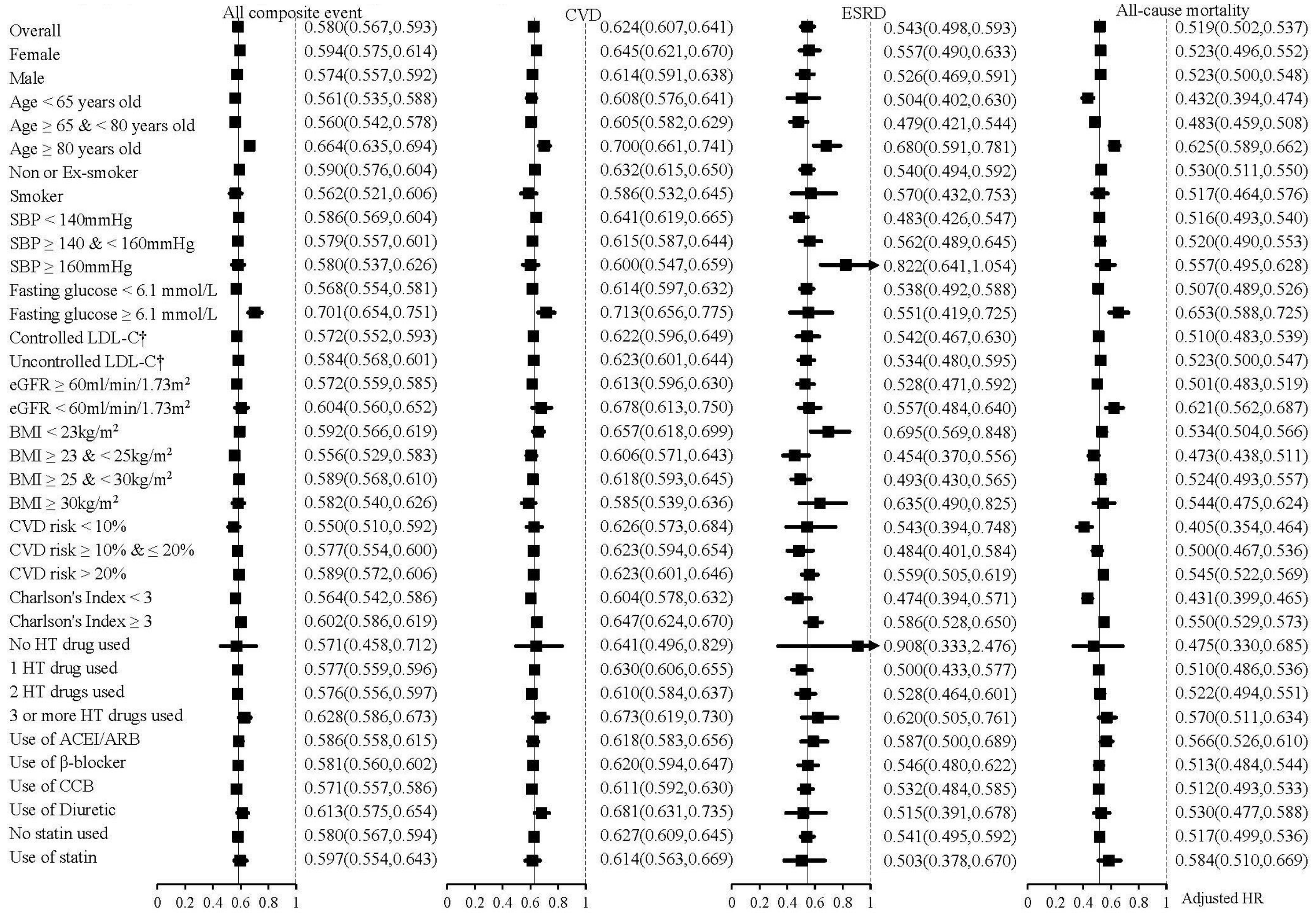

RAMP-HT = Risk Assessment and Management Program – Hypertension; SBP = Systolic Blood Pressure; LDL-C = Low Density Lipoprotein – Cholesterol; eGFR = estimated Glomerular Filtration Rate; BMI = Body Mass Index; CVD risk = Framingham 10-year CVD risk; HT = Hypertension; ACEI = Angiotensin Converting Enzyme Inhibitor; ARB = Angiotensin Receptor Blocker; CCB = Calcium Channel Blocker;
†Controlled LDL-C is defined as LDL-C < 3.4 mmol/L for CVD risk ≤ 20%, or LDL-C < 2.6 mmol/L for CVD risk > 20%, while uncontrolled LDL-C is defined as LDL-C ≥ 3.4 mmol/L for CVD risk ≤ 20%, or LDL-C ≥ 2.6 mmol/L for CVD risk > 20%.

© 2023 Yu EYT et al. *JAMA Network Open.*

eFigure 6. Adjusted incident rate ratios (IRRs) of RAMP-HT participants over usual care patients associated with the number of hospitalization, accident and emergency department (AED) attendance, special outpatient clinic (SOPC) and general outpatient clinic (GOPC) attendance in selected subgroups by negative binomial regressions. IRRs were adjusted for all covariates at baseline and the corresponding frequency of episode event within one year before baseline.

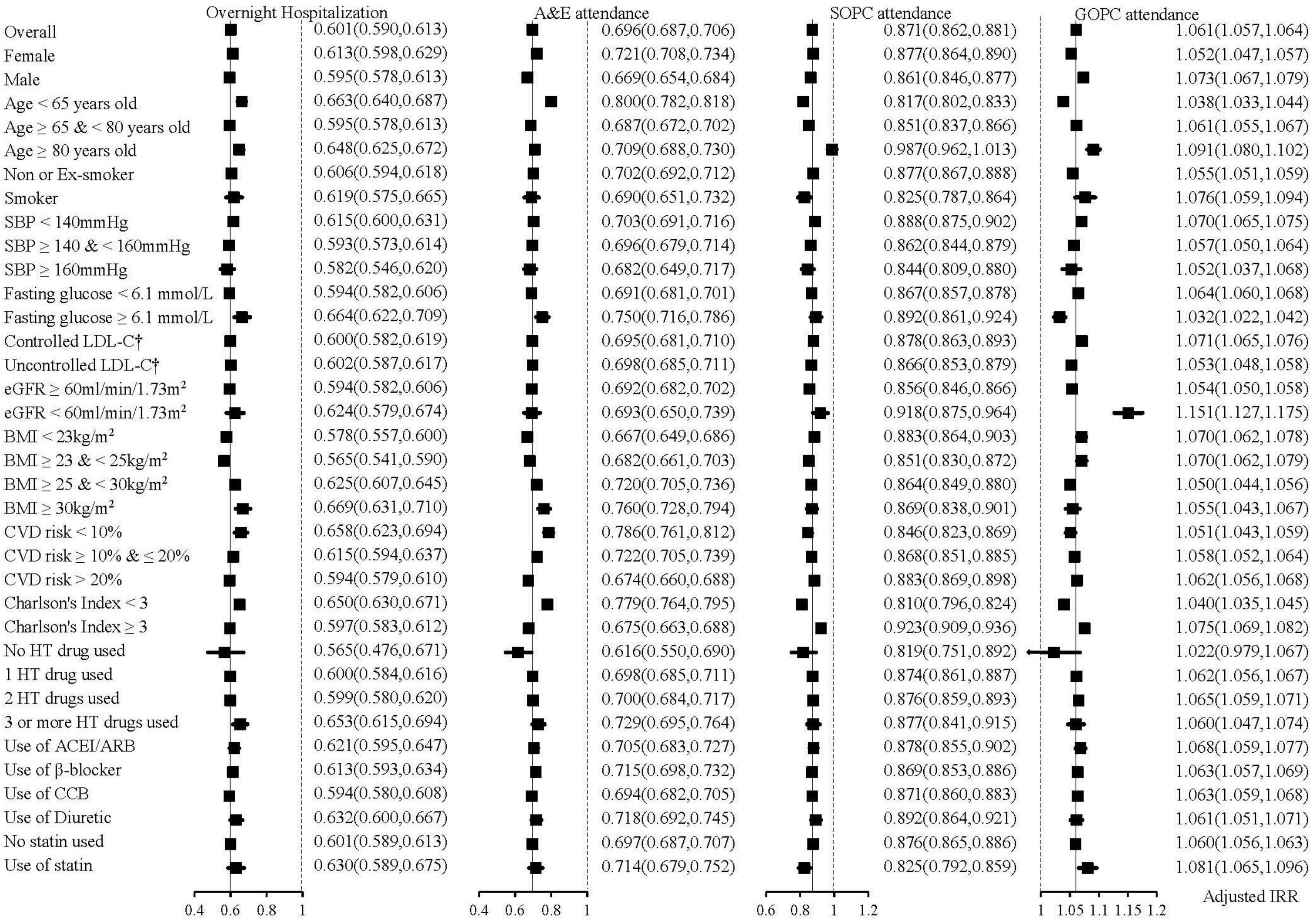

Subgroup	Overnight Hospitalization	A&E attendance	SOPC attendance	GOPC attendance
Overall	0.601(0.590,0.613)	0.696(0.687,0.706)	0.871(0.862,0.881)	1.061(1.057,1.064)
Female	0.613(0.598,0.629)	0.721(0.708,0.734)	0.877(0.864,0.890)	1.052(1.047,1.057)
Male	0.595(0.578,0.613)	0.669(0.654,0.684)	0.861(0.846,0.877)	1.073(1.067,1.079)
Age < 65 years old	0.663(0.640,0.687)	0.800(0.782,0.818)	0.817(0.802,0.833)	1.038(1.033,1.044)
Age ≥ 65 & < 80 years old	0.595(0.578,0.613)	0.687(0.672,0.702)	0.851(0.837,0.866)	1.061(1.055,1.067)
Age ≥ 80 years old	0.648(0.625,0.672)	0.709(0.688,0.730)	0.987(0.962,1.013)	1.091(1.080,1.102)
Non or Ex-smoker	0.606(0.594,0.618)	0.702(0.692,0.712)	0.877(0.867,0.888)	1.055(1.051,1.059)
Smoker	0.619(0.575,0.665)	0.690(0.651,0.732)	0.825(0.787,0.864)	1.076(1.059,1.094)
SBP < 140mmHg	0.615(0.600,0.631)	0.703(0.691,0.716)	0.888(0.875,0.902)	1.070(1.065,1.075)
SBP ≥ 140 & < 160mmHg	0.593(0.573,0.614)	0.696(0.679,0.714)	0.862(0.844,0.879)	1.057(1.050,1.064)
SBP ≥ 160mmHg	0.582(0.546,0.620)	0.682(0.649,0.717)	0.844(0.809,0.880)	1.052(1.037,1.068)
Fasting glucose < 6.1 mmol/L	0.594(0.582,0.606)	0.691(0.681,0.701)	0.867(0.857,0.878)	1.064(1.060,1.068)
Fasting glucose ≥ 6.1 mmol/L	0.664(0.622,0.709)	0.750(0.716,0.786)	0.892(0.861,0.924)	1.032(1.022,1.042)
Controlled LDL-C†	0.600(0.582,0.619)	0.695(0.681,0.710)	0.878(0.863,0.893)	1.071(1.065,1.076)
Uncontrolled LDL-C†	0.602(0.587,0.617)	0.698(0.685,0.711)	0.866(0.853,0.879)	1.053(1.048,1.058)
eGFR ≥ 60ml/min/1.73m^2	0.594(0.582,0.606)	0.692(0.682,0.702)	0.856(0.846,0.866)	1.054(1.050,1.058)
eGFR < 60ml/min/1.73m^2	0.624(0.579,0.674)	0.693(0.650,0.739)	0.918(0.875,0.964)	1.151(1.127,1.175)
BMI < 23kg/m^2	0.578(0.557,0.600)	0.667(0.649,0.686)	0.883(0.864,0.903)	1.070(1.062,1.078)
BMI ≥ 23 & < 25kg/m^2	0.565(0.541,0.590)	0.682(0.661,0.703)	0.851(0.830,0.872)	1.070(1.062,1.079)
BMI ≥ 25 & < 30kg/m^2	0.625(0.607,0.645)	0.720(0.705,0.736)	0.864(0.849,0.880)	1.050(1.044,1.056)
BMI ≥ 30kg/m^2	0.669(0.631,0.710)	0.760(0.728,0.794)	0.869(0.838,0.901)	1.055(1.043,1.067)
CVD risk < 10%	0.658(0.623,0.694)	0.786(0.761,0.812)	0.846(0.823,0.869)	1.051(1.043,1.059)
CVD risk ≥ 10% & ≤ 20%	0.615(0.594,0.637)	0.722(0.705,0.739)	0.868(0.851,0.885)	1.058(1.052,1.064)
CVD risk > 20%	0.594(0.579,0.610)	0.674(0.660,0.688)	0.883(0.869,0.898)	1.062(1.056,1.068)
Charlson's Index < 3	0.650(0.630,0.671)	0.779(0.764,0.795)	0.810(0.796,0.824)	1.040(1.035,1.045)
Charlson's Index ≥ 3	0.597(0.583,0.612)	0.675(0.663,0.688)	0.923(0.909,0.936)	1.075(1.069,1.082)
No HT drug used	0.565(0.476,0.671)	0.616(0.550,0.690)	0.819(0.751,0.892)	1.022(0.979,1.067)
1 HT drug used	0.600(0.584,0.616)	0.698(0.685,0.711)	0.874(0.861,0.887)	1.062(1.056,1.067)
2 HT drugs used	0.599(0.580,0.620)	0.700(0.684,0.717)	0.876(0.859,0.893)	1.065(1.059,1.071)
3 or more HT drugs used	0.653(0.615,0.694)	0.729(0.695,0.764)	0.877(0.841,0.915)	1.060(1.047,1.074)
Use of ACEI/ARB	0.621(0.595,0.647)	0.705(0.683,0.727)	0.878(0.855,0.902)	1.068(1.059,1.077)
Use of β-blocker	0.613(0.593,0.634)	0.715(0.698,0.732)	0.869(0.853,0.886)	1.063(1.057,1.069)
Use of CCB	0.594(0.580,0.608)	0.694(0.682,0.705)	0.871(0.860,0.883)	1.063(1.059,1.068)
Use of Diuretic	0.632(0.600,0.667)	0.718(0.692,0.745)	0.892(0.864,0.921)	1.061(1.051,1.071)
No statin used	0.601(0.589,0.613)	0.697(0.687,0.707)	0.876(0.865,0.886)	1.060(1.056,1.063)
Use of statin	0.630(0.589,0.675)	0.714(0.679,0.752)	0.825(0.792,0.859)	1.081(1.065,1.096)

RAMP-HT = Risk Assessment and Management Program – Hypertension; SBP = Systolic Blood Pressure; LDL-C = Low Density Lipoprotein – Cholesterol; eGFR = estimated Glomerular Filtration Rate; BMI = Body Mass Index; CVD risk = Framingham 10-year CVD risk; HT = Hypertension; ACEI = Angiotensin Converting Enzyme Inhibitor; ARB = Angiotensin Receptor Blocker; CCB = Calcium Channel Blocker;
†Controlled LDL-C is defined as LDL-C < 3.4 mmol/L for CVD risk ≤ 20%, or LDL-C < 2.6 mmol/L for CVD risk > 20%, while uncontrolled LDL-C is defined as LDL-C ≥ 3.4 mmol/L for CVD risk ≤ 20%, or LDL-C ≥ 2.6 mmol/L for CVD risk > 20%.

© 2023 Yu EYT et al. *JAMA Network Open.*

eTable 1. Definition of the event outcome measures

Event	ICPC-2 codes*	ICD-9-CM codes[#]	Clinical parameters
Hypertension	K86, K87	NA	NA
CHD	K74-K76	410-414	NA
Heart Failure	K77	428	NA
Stroke	K89-K91	430-438	NA
ESRD	NA	585.5, 585.6, 586	eGFR < 15ml/min/1.73m^2
DM	T89, T90	NA	NA

ICPC-2 = International Classification of Primary Care-2; ICD-9-CM = International Classification of Diseases, Ninth Edition, Clinical Modification; CHD = Coronary Heart Disease; ESRD = End Stage Renal Disease; eGFR = Estimated Glomerular Filtration Rate; DM = Diabetes Mellitus; NA = Not Applicable

*The ICPC-2 coding system is adopted in the public primary care clinics in Hong Kong to better capture the nature and variety of primary care patients' presenting problems.

[#]The ICD-9-CM coding system is used in the public hospital-based setting.

© 2023 Yu EYT et al. *JAMA Network Open.*

eTable 2. **Data completion rates of matched RAMP-HT participants and usual care patients at baseline and 5-year follow-up**

Time frame	At baseline		At 5 years	
**Factor**	RAMP-HT Participants (N = 108,045)	Usual Care Patients (N = 104,662)	RAMP-HT Participants (N = 108,045)	Usual Care Patients (N = 104,662)
**Socio-Demographic (n, %)**				
**Gender**	108,045 (100.00 %)	104,662 (100.00 %)	108,045 (100.00 %)	104,662 (100.00 %)
**Age**	108,045 (100.00 %)	104,662 (100.00 %)	108,045 (100.00 %)	104,662 (100.00 %)
**Smoking Status**	108,022 (99.98 %)	103,554 (98.94 %)	108,022 (99.98 %)	103,554 (98.94 %)
**Clinical Characteristics (n, %)**				
**SBP**	108,045 (100.00 %)	104,321 (99.67 %)	100,155 (92.70 %)	80.59 % (84,352)
**DBP**	108,045 (100.00 %)	104,321 (99.67 %)	100,155 (92.70 %)	80.59 % (84,344)
**Fasting Glucose**	107,787 (99.76 %)	89,943 (85.93 %)	96,653 (89.46 %)	75.61 % (79,130)
**LDL-C**	99.63 % (107,647)	87,696 (83.79 %)	97,900 (90.61 %)	76.06 % (79,602)
**TC/HDL-C Ratio**	107,891 (99.86 %)	88,044 (84.12 %)	98,026 (90.73 %)	76.15 % (79,695)
**Triglyceride**	107,917 (99.88 %)	88,577 (84.63 %)	98,035 (90.74 %)	76.22 % (79,778)
**BMI**	107,816 (99.79 %)	73,181 (69.92 %)	84,900 (78.58 %)	57.08 % (59,740)
**eGFR**	107,961 (99.92 %)	99,077 (94.66 %)	101,340 (93.79 %)	85.14 % (89,104)
**Charlson Comorbidity Index**	108,045 (100.00 %)	104,662 (100.00 %)	108,045 (100.00 %)	104,662 (100.00 %)
**Use of ACEI/ARB**	108,045 (100.00 %)	104,662 (100.00 %)	108,045 (100.00 %)	104,662 (100.00 %)
**Use of β-blocker**	108,045 (100.00 %)	104,662 (100.00 %)	108,045 (100.00 %)	104,662 (100.00 %)
**Use of CCB**	108,045 (100.00 %)	104,662 (100.00 %)	108,045 (100.00 %)	104,662 (100.00 %)
**Use of Diuretic**	108,045 (100.00 %)	104,662 (100.00 %)	108,045 (100.00 %)	104,662 (100.00 %)
**Use of other anti-hypertensive drugs**	108,045 (100.00 %)	104,662 (100.00 %)	108,045 (100.00 %)	104,662 (100.00 %)
**Use of Statin**	108,045 (100.00 %)	104,662 (100.00 %)	108,045 (100.00 %)	104,662 (100.00 %)
**Use of Fibrate**	108,045 (100.00 %)	104,662 (100.00 %)	108,045 (100.00 %)	104,662 (100.00 %)
**Frequency of Service Use†**				
**Overnight Hospitalization**	108,045 (100.00 %)	104,662 (100.00 %)	108,045 (100.00 %)	104,662 (100.00 %)
**Accident and Emergency Department**	108,045 (100.00 %)	104,662 (100.00 %)	108,045 (100.00 %)	104,662 (100.00 %)
**Specialist Out-patient Clinic**	108,045 (100.00 %)	104,662 (100.00 %)	108,045 (100.00 %)	104,662 (100.00 %)
**General Out-patient Clinic**	108,045 (100.00 %)	104,662 (100.00 %)	108,045 (100.00 %)	104,662 (100.00 %)

RAMP-HT = Risk Assessment and Management Program - Hypertension; SBP = Systolic Blood Pressure; DBP = Diastolic Blood Pressure; LDL-C = Low Density Lipoprotein - Cholesterol; TC = Total Cholesterol; HDL-C = High Density Lipoprotein - Cholesterol; BMI = Body Mass Index; eGFR = Estimated Glomerular Filtration Rate; ACEI = Angiotensin Converting Enzyme Inhibitor; ARB = Angiotensin Receptor Blocker; CCB = Calcium Channel Blocker

Notes:
†Service use was measured 1) at baseline: from a year before baseline to baseline; 2) at 5 years: from baseline to last follow-up

© 2023 Yu EYT et al. *JAMA Network Open.*

eTable 3. Baseline characteristics between RAMP-HT participants and usual care patients before and after multiple imputation without fine stratification weightings

	Before multiple imputation		After multiple imputation	
Factor	RAMP-HT Participants (N = 108,045)	Usual Care Patients (N = 104,671)	RAMP-HT Participants (N = 108,045)	Usual Care Patients (N = 104,671)
**Demographics (n, %)**				
Gender				
Female	64,050 (59.3 %)	59,466 (56.8 %)	59.3 % (64,050)	56.8 % (59,466)
Male	43,995 (40.7 %)	45,205 (43.2 %)	40.7 % (43,995)	43.2 % (45,205)
Age (mean±SD), year	65.1±10.8 (108,045)	68.1±13.5 (104,671)	65.1±10.8 (108,045)	68.1±13.5 (104,671)
Smoking Status				
Non-Smoker	100,876 (93.4 %)	94,616 (91.4 %)	93.4 % (100,897)	91.4 % (95,645)
Smoker	7,146 (6.6 %)	8,938 (8.6 %)	6.6 % (7,148)	8.6 % (9,026)
**Clinical Characteristics (mean±SD)**				
SBP, mm Hg	136.9±15.5 (108,045)	136.3±17.3 (104,321)	136.9±15.5 (108,045)	136.3±17.3 (104,671)
DBP, mm Hg	76.7±10.6 (108,045)	75.7±11.3 (104,321)	76.7±10.6 (108,045)	75.7±11.3 (104,671)
Fasting Glucose, mmol/L	5.3±0.6 (107,787)	5.4±0.8 (89,943)	5.3±0.6 (108,045)	5.4±0.8 (104,671)
LDL-C, mmol/L	3.2±0.8 (107,647)	3.2±0.8 (87,696)	3.2±0.8 (108,045)	3.2±0.9 (104,671)
TC/HDL-C Ratio	3.9±1.2 (107,891)	4.0±2.7 (88,046)	3.9±1.2 (108,045)	4.0±2.9 (104,671)
Triglyceride, mmol/L	1.4±0.9 (107,917)	1.5±0.9 (88,577)	1.4±0.9 (108,045)	1.5±1.0 (104,671)
BMI, kg/m^2	25.6±3.9 (107,816)	25.3±4.1 (73,181)	25.6±3.9 (108,045)	25.2±5.2 (104,671)
eGFR (n, %)				
≥ 60ml/min/1.73m^2	104,870 (97.1 %)	92,615 (93.5 %)	97.1 % (104,948)	93.5 % (97,867)
< 60ml/min/1.73m^2	3,091 (2.9 %)	6,462 (6.5 %)	2.9 % (3,097)	6.5 % (6,804)
Charlson Comorbidity Index	3.0±1.2 (108,045)	3.2±1.3 (104,671)	3.0±1.2 (108,045)	3.2±1.3 (104,671)
Number of HT drugs used (n, %)				
0	1,166 (1.1 %)	4,103 (3.9 %)	1,166 (1.1 %)	4,103 (3.9 %)
1	57,819 (53.5 %)	56,446 (53.9 %)	57,819 (53.5 %)	56,446 (53.9 %)
2	40,011 (37.0 %)	36,005 (34.4 %)	40,011 (37.0 %)	36,005 (34.4 %)
≥3	9,049 (8.4 %)	8,117 (7.8 %)	9,049 (8.4 %)	8,117 (7.8 %)
Use of ACEI/ARB (n, %)	22,480 (20.8 %)	19,013 (18.2 %)	22,480 (20.8 %)	19,013 (18.2 %)
Use of β-blocker (n, %)	41,632 (38.5 %)	37,063 (35.4 %)	41,632 (38.5 %)	37,063 (35.4 %)
Use of CCB (n, %)	78,260 (72.4 %)	71,649 (68.5 %)	78,260 (72.4 %)	71,649 (68.5 %)
Use of Diuretic (n, %)	12,762 (11.8 %)	13,679 (13.1 %)	12,762 (11.8 %)	13,679 (13.1 %)
Use of other anti-hypertensive drugs (n, %)	10,785 (10.0 %)	12,250 (11.7 %)	10,785 (10.0 %)	12,250 (11.7 %)
Use of Statin (n, %)	9,460 (8.8 %)	6,651 (6.4 %)	9,460 (8.8 %)	6,651 (6.4 %)
Use of Fibrate (n, %)	2,060 (1.9 %)	1,648 (1.6 %)	2,060 (1.9 %)	1,648 (1.6 %)
**Frequency of Service Use† (mean±SD)**				
Overnight Hospitalization	0.1±0.5 (108,045)	0.2±0.8 (104,671)	0.1±0.5 (108,045)	0.2±0.8 (104,671)
Accident & Emergency Department	0.3±0.9 (108,045)	0.5±1.5 (104,671)	0.3±0.9 (108,045)	0.5±1.5 (104,671)
Specialist Out-patient Clinic	1.5±2.7 (108,045)	1.8±3.2 (104,671)	1.5±2.7 (108,045)	1.8±3.2 (104,671)
General Out-patient Clinic	5.5±2.3 (108,045)	5.8±3.1 (104,671)	5.5±2.3 (108,045)	5.8±3.1 (104,671)

RAMP-HT = Risk Assessment and Management Program - Hypertension; SBP = Systolic Blood Pressure; DBP = Diastolic Blood Pressure; LDL-C = Low Density Lipoprotein - Cholesterol; TC = Total Cholesterol; HDL-C = High Density Lipoprotein - Cholesterol; BMI = Body Mass Index; eGFR = Estimated Glomerular Filtration Rate; ACEI = Angiotensin Converting Enzyme Inhibitor; ARB = Angiotensin Receptor Blocker; CCB = Calcium Channel Blocker; SD = Standard Deviation

Notes:
† Service use was measured from a year before baseline to baseline

© 2023 Yu EYT et al. *JAMA Network Open.*

eTable 4. Frequency of RAMP-HT service attendances among RAMP-HT participants

	RAMP-HT Participants (N = 108,045)		
	**Nurse assessment**	**Nurse intervention**	**Doctor intervention**
**Frequency (n, %)**			
1	40,031 (37.1 %)	13,823 (12.8 %)	1,291 (1.2 %)
2	37,422 (34.6 %)	3,170 (2.9 %)	821 (0.8 %)
3	18,008 (16.7 %)	1,153 (1.1 %)	546 (0.5 %)
4	7,152 (6.6 %)	559 (0.5 %)	307 (0.3 %)
≥ 5	5,432 (5.0 %)	633 (0.5 %)	505 (0.5 %)
**Mean±SD**	2.1±1.1	0.3±0.8	0.1±0.6

RAMP-HT = Risk Assessment and Management Program – Hypertension; SD = Standard deviation

© 2023 Yu EYT et al. *JAMA Network Open.*

eTable 5. Patients' characteristics between RAMP-HT participants and usual care patients at 5-year follow-up after fine stratification weightings

Characteristics	At 5 year		
	RAMP-HT Participants (N = 108,045)	Usual Care Patients (N = 104,662)	SMD
**Socio-Demographic (n, %)**			
Gender			0.003
Female	62,277 (57.6 %)	60,497 (57.8%)	
Age (mean±SD), year	71.3±12.3	71.3±13.5	0.003
Smoking Status			0.009
Smoker	6,913 (6.4 %)	6,858 (6.6%)	
**Clinical Characteristics (mean±SD)**			
SBP, mm Hg	132.5±16.3	134.7±17.1	0.143
DBP, mm Hg	73.5±11.4	74.5±11.3	0.090
Fasting Glucose, mmol/L	5.5±1.0	5.7±1.1	0.144
LDL-C, mmol/L	2.7±0.8	2.8±0.9	0.118
TC/HDL-C Ratio	3.6±1.7	3.7±1.2	0.079
Triglyceride, mmol/L	1.4±0.8	1.4±0.9	0.018
BMI, kg/m^2	25.5±4.2	25.5±4.5	0.016
eGFR (%, n)			0.083
≥ 60ml/min/1.73m^2	94,563 (93.3 %)	81,150 (91.1%)	
< 60ml/min/1.73m^2	6,777 (6.7 %)	7,947 (8.9%)	
Charlson Comorbidity Index	3.9±1.6	4.1±1.7	0.109
Number of HT drugs used (n, %)			0.025
0-1	56,703 (50.9 %)	50,317 (49.7 %)	
≥2	51,342 (49.1 %)	49.7 % (50.3 %)	
Use of ACEI/ARB (n, %)	34,452 (31.9 %)	33,429 (31.9%)	0.001
Use of β-blocker (n, %)	38,289 (35.4 %)	37,593 (35.9%)	0.01
Use of CCB (n, %)	81,908 (75.8 %)	77,925 (74.5%)	0.031
Use of Diuretic (n, %)	9,542 (8.8 %)	9,640 (9.2%)	0.013
Use of other anti-hypertensive drugs (n, %)	11,661 (10.8 %)	10,651 (10.2%)	0.020
Use of statin (n, %)	42,530 (39.4 %)	34,395 (32.9%)	0.136
Use of fibrate (n, %)	3,093 (2.9 %)	3,048 (2.9%)	0.003
**Frequency of Service Use† (mean±SD)**			
Overnight Hospitalization	1.1±2.9	1.9±3.3	0.270
Accident & Emergency Department	1.9±5.4	2.8±4.9	0.199
Specialist Out-patient Clinic	9.0±13.1	10.9±13.9	0.143
General Out-patient Clinic	21.0±13.9	22.3±14.0	0.099

RAMP-HT = Risk Assessment and Management Program - Hypertension; SBP = Systolic Blood Pressure; DBP = Diastolic Blood Pressure; LDL-C = Low Density Lipoprotein - Cholesterol; TC = Total Cholesterol; HDL-C = High Density Lipoprotein - Cholesterol; BMI = Body Mass Index; eGFR = Estimated Glomerular Filtration Rate; ACEI = Angiotensin Converting Enzyme Inhibitor; ARB = Angiotensin Receptor Blocker; CCB = Calcium Channel Blocker; SD = Standard Deviation, SMD = standardized mean difference

Notes:
†Uses of service use were measured from baseline
SMD<0.2 indicates balance between groups.

© 2023 Yu EYT et al. *JAMA Network Open.*

eTable 6. Sensitivity analyses on comparisons of all outcome event, cardiovascular disease, end stage renal disease, diabetes mellitus, and all-cause mortality between RAMP-HT participants and usual care patients

	Sensitivity analyses																							
	(1)			(2)			(3)			(4)			(5)			(6)			(7)			(8)		
Event	ARR	HR†	NNT	ARR	HR†	NNT	ARR	HR†	NNT	ARR	HR†	NNT	ARR	HR†	NNT	ARR	HR†	NNT	ARR	HR†	NNT	ARR	HR†	NNT
All outcome event	12.0%	0.61*	10	9.5%	0.59*	12	14.5%	0.55*	9	12.0 %	0.59*	12	9.2%	0.60*	12	12.0%	0.57*	10	14.5%	0.58*	11	14.5%	0.58*	11
CVD	6.4%	0.66*	23	5.8%	0.62*	17	8.0%	0.59*	15	7.4%	0.61*	17	5.8%	0.62*	17	7.4%	0.59*	15	8.0%	0.62*	16	8.0%	0.62*	16
CHD	1.9%	0.71*	65	2.0%	0.66*	47	2.6%	0.64*	41	2.6%	0.65*	46	2.1%	0.66*	46	2.6%	0.63*	41	2.6%	0.66*	47	2.6%	0.66*	47
Heart Failure	2.5%	0.56*	62	2.1%	0.54*	52	3.2%	0.50*	38	2.7%	0.53*	53	2.2%	0.53*	49	2.7%	0.51*	44	3.2%	0.54*	49	3.2%	0.54*	49
Stroke	3.2%	0.67*	80	3.0%	0.63*	35	4.0%	0.61*	30	3.7%	0.63*	36	2.8%	0.64*	36	3.7%	0.61*	32	4.0%	0.64*	36	4.0%	0.64*	36
ESRD	1.3%	0.54*	95	0.7%	0.62*	155	1.6%	0.50*	79	1.4%	0.54*	119	0.7%	0.63*	158	1.3%	0.52*	93	1.6%	0.54*	105	1.6%	0.54*	105
DM	4.3%	0.78*	91	4.0%	0.79*	33	4.6%	0.70*	23	6.3%	0.76*	28	4.5%	0.78*	30	6.3%	0.67*	19	4.6%	0.82*	41	4.6%	0.83*	41
All-Cause Mortality	8.2%	0.55*	21	6.0%	0.54*	20	10.0%	0.48*	14	7.4%	0.55*	22	5.6%	0.57*	22	7.4%	0.53*	18	10.0%	0.52*	17	10.0%	0.52*	17
CVD Mortality	3.0%	0.55*	61	2.4%	0.50*	47	3.9%	0.46*	32	3.1%	0.51*	51	2.3%	0.53*	50	3.1%	0.49*	41	3.9%	0.51*	43	3.9%	0.51*	43
Non-CVD Mortality	5.3%	0.55*	62	3.5%	0.56*	32	6.1%	0.49*	21	4.3%	0.58*	59	3.3%	0.59*	35	4.3%	0.56*	30	6.0%	0.53*	45	6.0%	0.53*	45

(1) = Excluded patients with less than 1-year follow-up; (2) = Multiple imputation with one-to-one propensity score matching; (3) = Multiple imputation without propensity score matching or weightings; (4) = Fine stratification weightings without multiple imputation; (5) = Propensity score matching only without multiple imputation; (6) = Complete case analysis; (7) = 25 Multiple imputations with propensity score fine stratification weightings; (8) = 50 Multiple imputations with propensity score fine stratification weightings

RAMP-HT = Risk Assessment and Management Program - Hypertension; CVD = Cardiovascular Disease; CHD = Coronary Heart Disease; ESRD = End Stage Renal Disease; DM = Diabetes Mellitus; ARR = Absolute Risk Reduction; HR = Hazard Ratio; NNT = Number Needed to Treat;

Notes:
* P-value <0.05.
† Hazard ratios were adjusted by gender, age, smoking status, SBP, DBP, fasting glucose, LDL-C, TC/HDL-C ratio, triglyceride, BMI, eGFR, Charlson Comorbidity Index and the usages of ACEI/ARB, β-blocker, CCB, Diuretic, other anti-hypertensive drugs, statin and fibrate at baseline by multivariable Cox proportional hazard regression/multivariable Cox proportional hazard regression with fine stratification weighting, as appropriate

© 2023 Yu EYT et al. *JAMA Network Open.*

eTable 7. Sensitivity analyses on comparisons of service use between RAMP-HT participants and usual care patients

	**Sensitivity analyses**															
	**(1)**		**(2)**		**(3)**		**(4)**		**(5)**		**(6)**		**(7)**		**(8)**	
**Frequency of Event during the Period**	**Rate**	**IRR†**	**Rate**	**IRR†**	**Rate**	**IRR†**	**Rate**	**IRR†**	**Rate**	**IRR†**	**Rate**	**IRR†**	**Rate**	**IRR†**	**Rate**	**IRR†**
**Overnight Hospitalization**	-12.4	0.62*	-12.6	0.61*	-21.7	0.57*	-17.0	0.62*	-12.1	0.62*	-17.2	0.59*	-13.3	0.60*	-13.3	0.60*
**AED Attendance**	-13.1	0.72*	-13.6	0.70*	-24.6	0.68*	-19.8	0.72*	-13.5	0.72*	-20.1	0.70*	-14.1	0.70*	-14.0	0.70*
**SOPC Attendance**	-17.3	0.88*	-23.5	0.85*	-39.7	0.85*	-41.2	0.85*	-22.5	0.85*	-42.6	0.83*	-18.1	0.87*	-18.1	0.87*
**GOPC Attendance**	14.6	1.05*	0.3	1.05*	9.2	1.06*	-5.4	1.04*	-1.7	1.04*	-8.5	1.04*	15.8	1.06*	15.7	1.06*

(1) = Excluded patients with less than 1-year follow-up; (2) = Multiple imputation with one-to-one propensity score matching; (3) = Multiple imputation without propensity score matching or weightings; (4) = Fine stratification weightings without multiple imputation; (5) = Propensity score matching only without multiple imputation; (6) = Complete case analysis; (7) = 25 Multiple imputations with propensity score fine stratification weightings; (8) = 50 Multiple imputations with propensity score fine stratification weightings

RAMP-HT = Risk Assessment and Management Program - Hypertension; AED = Accident and Emergency Department; SOPC = Specialist Outpatient Clinic; GOPC = General Outpatient Clinic; IRR = Incidence Rate Ratio

Notes:
The unit for incidence rate is Cases/100 Person Years.
* P-value <0.05.
† All incidence rate ratios were adjusted by gender, age, smoking status, SBP, DBP, fasting glucose, LDL-C, TC/HDL-C ratio, triglyceride, BMI, eGFR, Charlson Comorbidity Index and the usages of ACEI/ARB, β-blocker, CCB, Diuretic, other anti-hypertensive drugs, statin and fibrate, and the corresponding number of service use at baseline by multivariable negative binomial regression/multivariable negative binomial regression with fine stratification weighting, as appropriate

© 2023 Yu EYT et al. *JAMA Network Open.*
