# Supplementary material for: Assessment of Hypertension Complications and Health Service Use 5 Years After Implementation of a Multicomponent Intervention
Source: JAMA Netw Open. 2023 May 24;6(5):e2315064. doi: 10.1001/jamanetworkopen.2023.15064 (PMC10209746; doi:10.1001/jamanetworkopen.2023.15064)
Supplement: Supplement 2. — Data Sharing Statement [file jamanetwopen-e2315064-s002.pdf]

Data Sharing Statement

Yu. Assessment of Hypertension Complications and Health Service Use 5 Years After Implementation of a Multicomponent Intervention. *JAMA Netw Open*. Published May 24, 2023. doi:10.1001/jamanetworkopen.2023.15064

Data
Data available: No

Additional Information
Explanation for why data not available: As this study is based on data from the Hong Kong Hospital Authority (HA), the authors are not allowed to share the data due to concerns with patient confidentiality based on an agreement with the HA.
